# Supplementary material for: Prenatal and early postnatal periods differentially shape the maturation of human cortical microstructure and myelin
Source: PLoS Biol. 2026 Mar 26;24(3):e3003722. doi: 10.1371/journal.pbio.3003722 (PMC13046243; doi:10.1371/journal.pbio.3003722)
Supplement: S1 Fig — Linear regression models were used to assess the association between postmenstrual age (PMA) and intracortical profile moments across cortical regions, while controlling for sex. Bigger surface maps were derived from a subsample of participants, which excluded any participants of multiple pregnancies. Smaller surface maps were derived from the original sample of this study. All surface maps display t-values for the PMA-estimate, projected onto the cortical surface for center of gravity (top) and variance (bottom). The spatial correlation between the effects on the original dataset and the dataset excluding twins is displayed in the middle of each set, together with the p-value derived from spin-based permutation testing (n = 10,000), between the 2.5th to 97.5th percentile of the permuted correlations. Excluded parcels are displayed in gray. (PDF) [file pbio.3003722.s001.pdf]

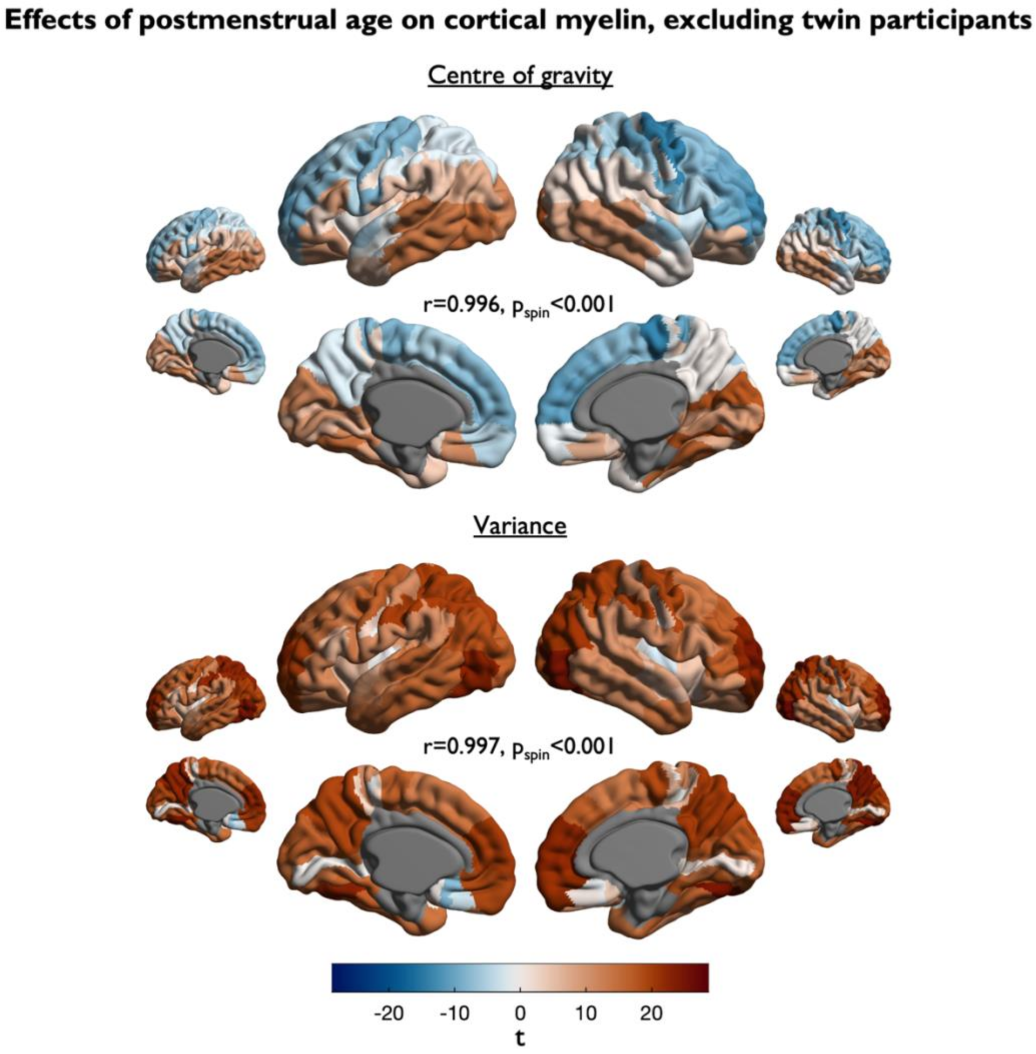

S1 Fig: Linear regression models were used to assess the association between postmenstrual age (PMA) and intracortical profile moments across cortical regions, while controlling for sex. Bigger surface maps were derived from a subsample of participants, which excluded any participants of multiple pregnancies. Smaller surface maps were derived from the original sample of this study. All surface maps display t-values for the PMA-estimate, projected onto the cortical surface for centre of gravity (top) and variance (bottom). The spatial correlation between the effects on the original dataset and the dataset excluding twins is displayed in the middle of each set, together with the p-value derived from spin-based permutation testing (n = 10000), between the 2.5th to 97.5th percentile of the permuted correlations. Excluded parcels are displayed in grey.
