# Supplementary material for: Prenatal and early postnatal periods differentially shape the maturation of human cortical microstructure and myelin
Source: PLoS Biol. 2026 Mar 26;24(3):e3003722. doi: 10.1371/journal.pbio.3003722 (PMC13046243; doi:10.1371/journal.pbio.3003722)
Supplement: S4 Fig — Linear regression models were used to asses the association between postmenstrual (left), gestational (middle) and postnatal age (right) and intracortical profile moments across cortical regions, while excluding extremely preterm (EPT) infants from the analyses. The first and third rows of surface maps display the t-values for the developmental variables in the original dataset with all participants, whereas the second and forth rows display the t-values for the developmental variables in the subset excluding EPT participants. The first two rows correspond to the linear models with center of gravity as response variable, whereas the third and forth rows correspond to the models with variance as response variable. Excluded parcels are displayed in gray. (PDF) [file pbio.3003722.s004.pdf]

Effects on cortical myelin, excluding extremely preterm infants

Centre of gravity, μ1

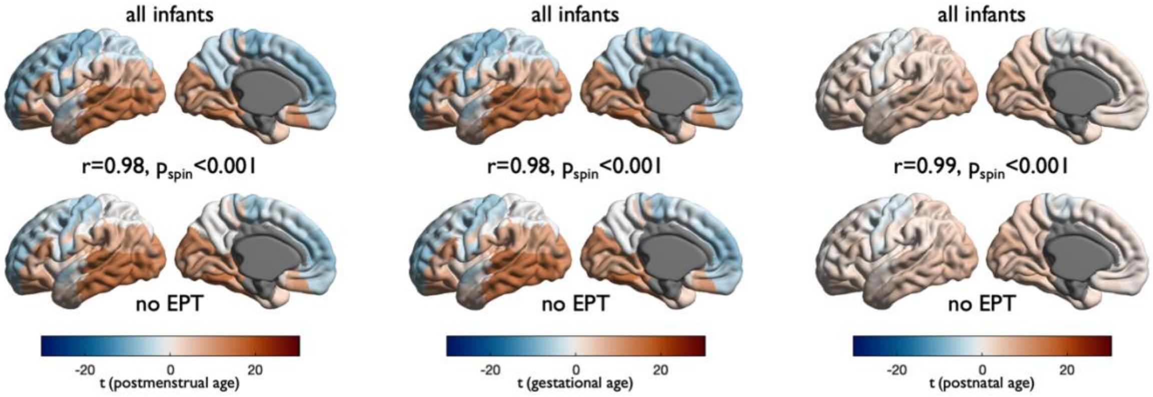

Variance, μ2

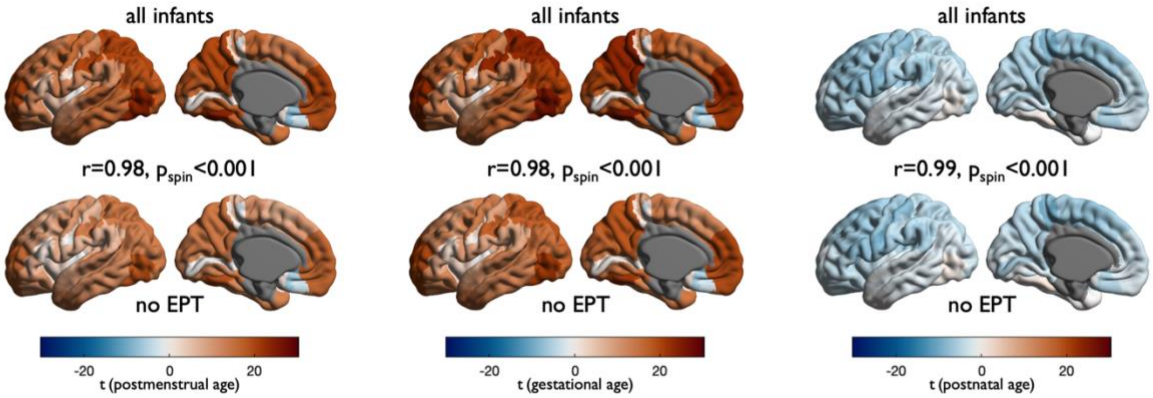

S4 Fig: Linear regression models were used to asses the association between postmenstrual (left), gestational (middle) and postnatal age (right) and intracortical profile moments across cortical regions, while excluding extremely preterm (EPT) infants from the analyses. The first and third rows of surface maps display the t-values for the developmental variables in the original dataset with all participants, whereas the second and forth rows display the t-values for the developmental variables in the subset excluding EPT participants. The first two rows correspond to the linear models with centre of gravity as response variable, whereas the third and forth rows correspond to the models with variance as response variable. Excluded parcels are displayed in grey.
