# Supplementary material for: Prenatal and early postnatal periods differentially shape the maturation of human cortical microstructure and myelin
Source: PLoS Biol. 2026 Mar 26;24(3):e3003722. doi: 10.1371/journal.pbio.3003722 (PMC13046243; doi:10.1371/journal.pbio.3003722)
Supplement: S7 Fig — Linear regression models were used to assess the association between postmenstrual age (PMA) and intracortical profile moments across cortical regions of the Schaefer-200 atlas, while controlling for sex. Bigger surface maps display t-values for the PMA-estimate, projected onto the cortical surface for center of gravity (top) and variance (bottom). Smaller surface maps show the results of the same models using the von Economo atlas. The spatial correlation between the effects on the Schaefer-200 and von Economo atlas is displayed in the middle of each set, together with the p-value derived from spin-based permutation testing (n = 10,000), between the 2.5th to 97.5th percentile of the permuted correlations. To calculate these correlations, parcel-wise results were upsampled to vertex-wise level. Excluded parcels are displayed in gray. (PDF) [file pbio.3003722.s007.pdf]

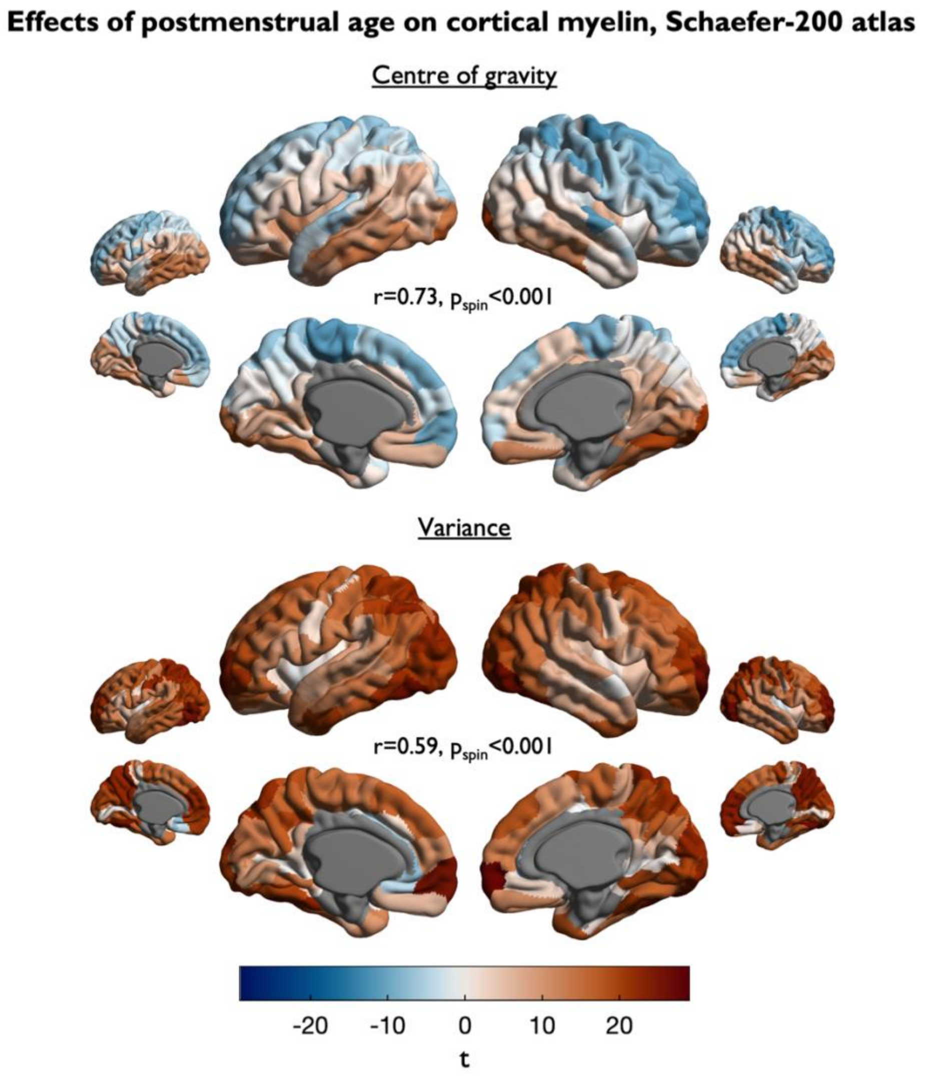

S7 Fig: Linear regression models were used to assess the association between postmenstrual age (PMA) and intracortical profile moments across cortical regions of the Schaefer-200 atlas, while controlling for sex. Bigger surface maps display t-values for the PMA-estimate, projected onto the cortical surface for centre of gravity (top) and variance (bottom). Smaller surface maps show the results of the same models using the von Economo atlas. The spatial correlation between the effects on the Schaefer-200 and von Economo atlas is displayed in the middle of each set, together with the p-value derived from spin-based permutation testing (n = 10000), between the 2.5th to 97.5th percentile of the permuted correlations. To calculate these correlations, parcel-wise results were upsampled to vertex-wise level. Excluded parcels are displayed in grey.
