# Supplementary material for: Prenatal and early postnatal periods differentially shape the maturation of human cortical microstructure and myelin
Source: PLoS Biol. 2026 Mar 26;24(3):e3003722. doi: 10.1371/journal.pbio.3003722 (PMC13046243; doi:10.1371/journal.pbio.3003722)
Supplement: S10 Fig — Parcel-wise T1w/T2w intensity distributions mapped on the dHCP 40-week surface template. Excluded regions (i.e., von Economo areas LA1, LA2, LC1, LC2, LC3, LD, and LE and the cortical wall) are shown in gray. (PDF) [file pbio.3003722.s010.pdf]

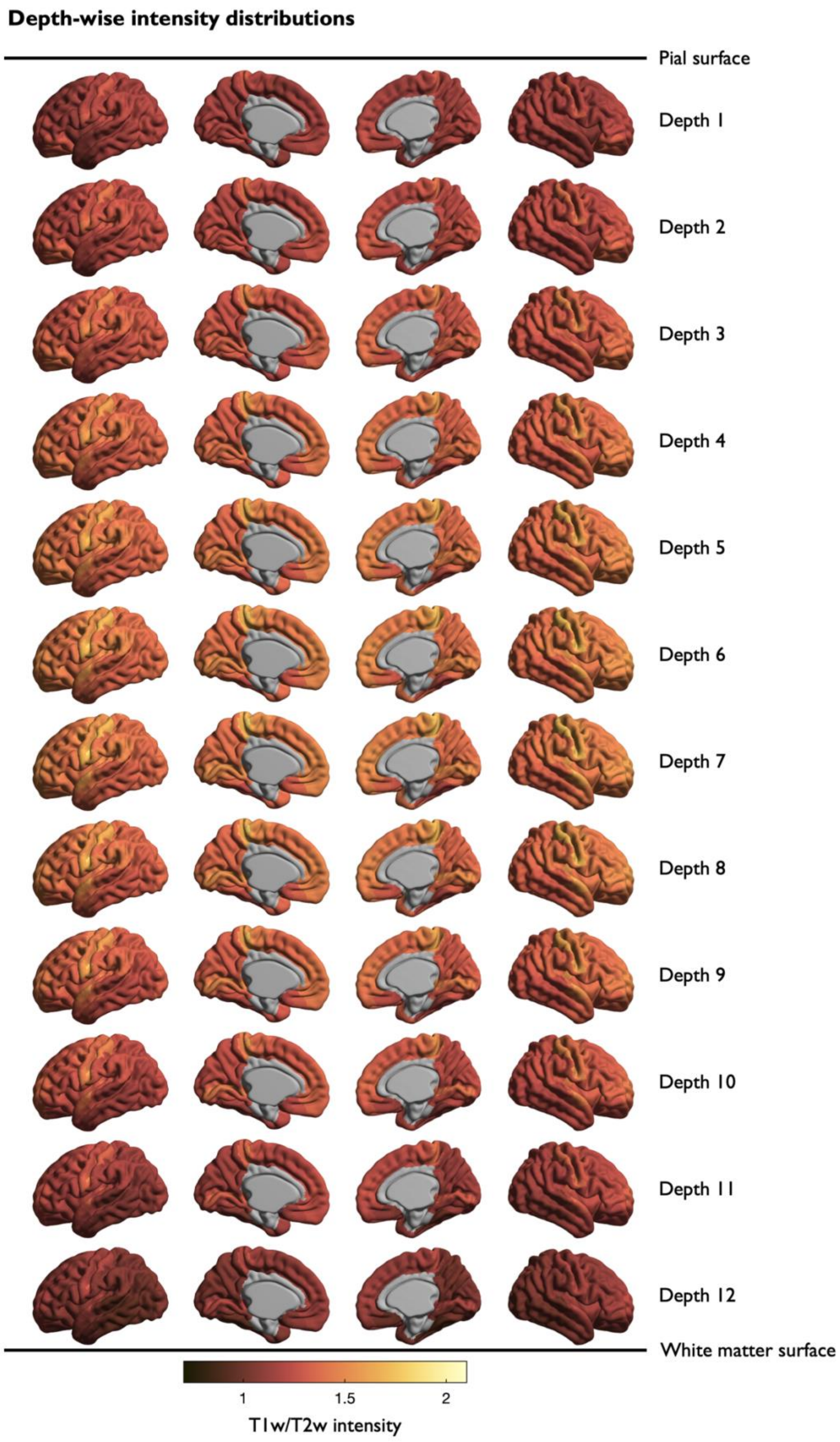

S10 Fig: Parcel-wise T1w/T2w intensity distributions mapped on the dHCP 40-week surface template. Excluded regions (i.e. von Economo areas L_{A1}, L_{A2}, L_{C1}, L_{C2}, L_{C3}, L_D and L_E and the cortical wall) are shown in grey.
