# Supplementary material for: Prenatal and early postnatal periods differentially shape the maturation of human cortical microstructure and myelin
Source: PLoS Biol. 2026 Mar 26;24(3):e3003722. doi: 10.1371/journal.pbio.3003722 (PMC13046243; doi:10.1371/journal.pbio.3003722)
Supplement: S11 Fig — Cortical surface maps illustrate the spatial distribution of gestational (left) and postnatal (right) age effects on each central moment, without controlling for postnatal and gestational age, respectively. The parcel-wise estimates were obtained from linear regression models with gestational age and sex or postnatal age and sex as predictors. Excluded parcels are displayed in gray. (PDF) [file pbio.3003722.s011.pdf]

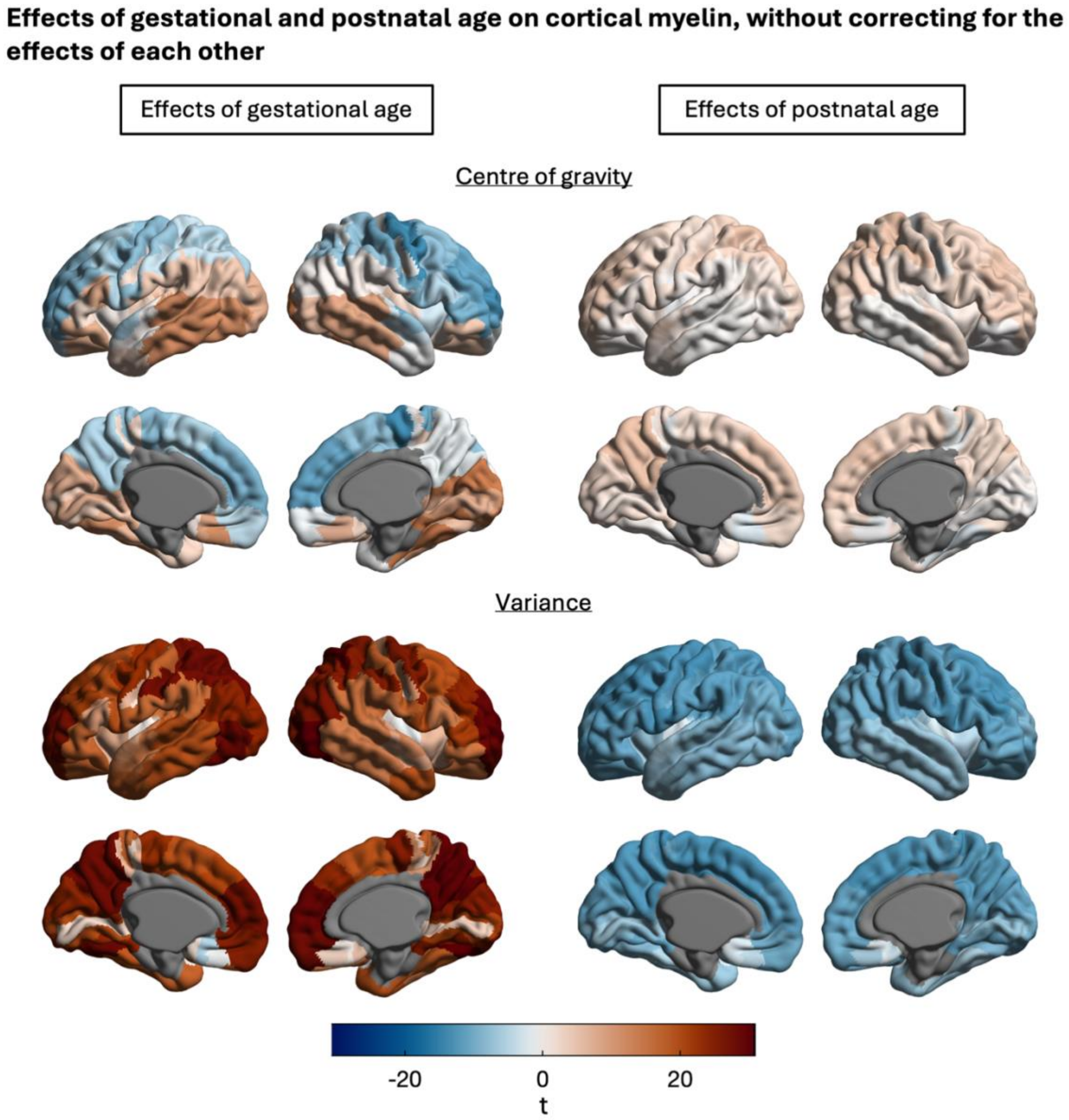

S11 Fig: Cortical surface maps illustrate the spatial distribution of gestational (left) and postnatal (right) age effects on each central moment, without controlling for postnatal and gestational age respectively. The parcel-wise estimates were obtained from linear regression models with gestational age and sex or postnatal age and sex as predictors. Excluded parcels are displayed in grey.
