# Supplementary material for: Prenatal and early postnatal periods differentially shape the maturation of human cortical microstructure and myelin
Source: PLoS Biol. 2026 Mar 26;24(3):e3003722. doi: 10.1371/journal.pbio.3003722 (PMC13046243; doi:10.1371/journal.pbio.3003722)
Supplement: S13 Fig — Linear regression models were used to assess the association between gestational age (GA) and intracortical profile moments across cortical regions, while controlling for cortical thickness and sex. The original results of this study, without controlling for cortical thickness are displayed as smaller surface maps. All surface maps display t-values for the GA-estimate, projected onto the cortical surface for center of gravity (top) and variance (bottom). The spatial correlation between the effects with and without controlling for cortical thickness is displayed in the middle of each set, together with the p-value derived from spin-based permutation testing (n = 10,000), between the 2.5th to 97.5th percentile of the permuted correlations. Excluded parcels are displayed in gray. (PDF) [file pbio.3003722.s013.pdf]

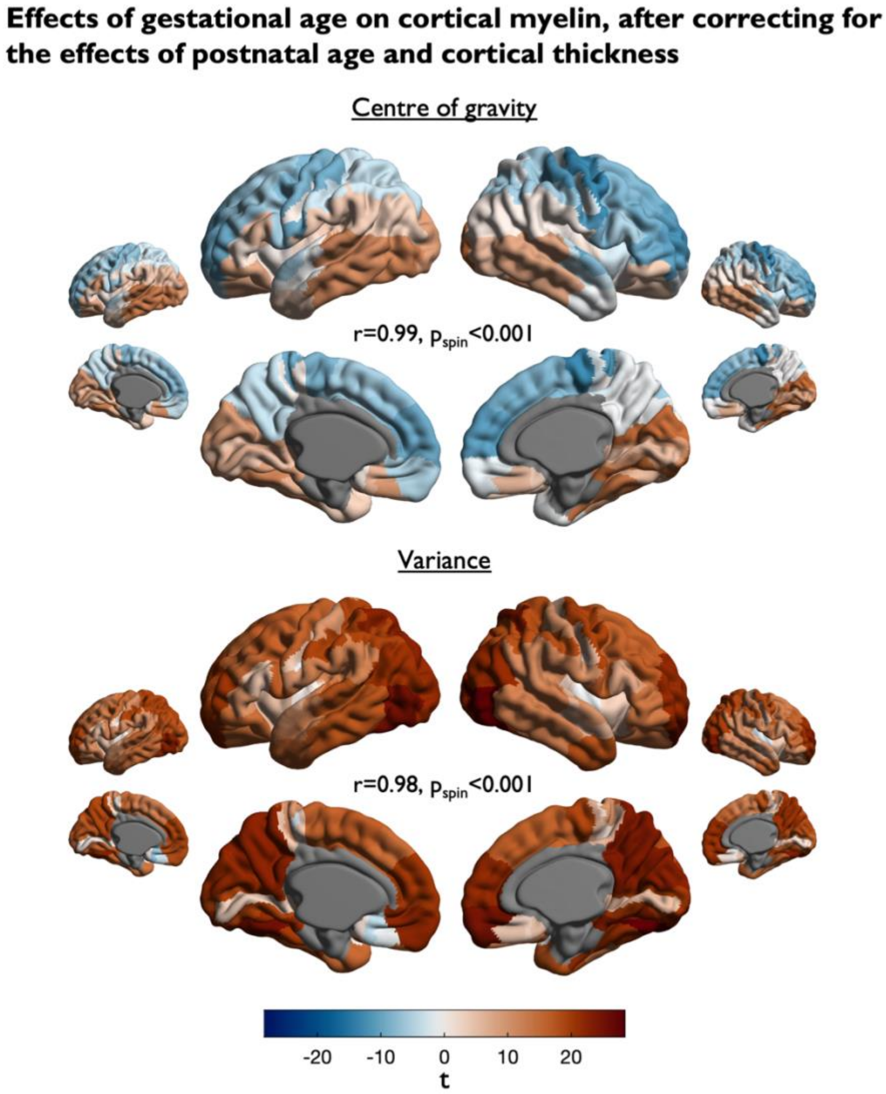

S13 Fig: Linear regression models were used to assess the association between gestational age (GA) and intracortical profile moments across cortical regions, while controlling for cortical thickness and sex. The original results of this study, without controlling for cortical thickness are displayed as smaller surface maps. All surface maps display t-values for the GA-estimate, projected onto the cortical surface for centre of gravity (top) and variance (bottom). The spatial correlation between the effects with and without controlling for cortical thickness is displayed in the middle of each set, together with the p-value derived from spin-based permutation testing (n = 10000), between the 2.5th to 97.5th percentile of the permuted correlations. Excluded parcels are displayed in grey.
