# Supplementary material for: Prenatal and early postnatal periods differentially shape the maturation of human cortical microstructure and myelin
Source: PLoS Biol. 2026 Mar 26;24(3):e3003722. doi: 10.1371/journal.pbio.3003722 (PMC13046243; doi:10.1371/journal.pbio.3003722)
Supplement: Attachment — Submitted filename: Response_to_reviewers_060226.pdf [file pbio.3003722.s016.pdf]

We would like to thank the Editors for the opportunity to submit a revised manuscript and are grateful to have received constructive comments from the Reviewers. Following their suggestions, we believe the quality of our paper has significantly improved, especially in terms of conceptual clarity and the discussion of methodological limitations. We have addressed all Reviewer comments in a point-by-point fashion below and highlighted the corresponding changes in the manuscript in red.

Reviewer 1 (R1):

R1 summary: The manuscript presents a novel analysis of neonatal intracortical microstructure. Using a very large dataset of anatomical infant MRI, the authors sampled T1w/T2w ratios across equidistant surfaces spanning the cortical depth to model signal variation from pial to white matter. Profiles were summarized by mean intensity, centre of gravity, and variance, and developmental trajectories were examined with respect to gestational and postnatal age, then related to low spatial-frequency geometric eigenmodes. The approach of probing within-tissue properties and sublaminar features is interesting and potentially valuable. The study is generally well organized and clearly written. However, I have several concerns, especially regarding methodological limitations inherent to infant imaging and profile-based analyses, and some interpretations.

We would like to sincerely thank Reviewer 1 for their positive evaluation of our work and for providing such constructive and helpful feedback.

R1#1: T1w/T2w ratio interpretation and terminology

Terms such as 'microstructural density' or 'increase in microstructure' are imprecise. Microstructure is not a direct metric; please describe the actual measured quantities (mean T1w/T2w values, variance etc). I'm not sure I understand what 'microstructural density' means in this context.

Framing of T1w/T2w as a direct measure of microstructure should be interpreted with caution. Actually, you provide more details in Methods when describing the T1w/T2w, some of it repeats the introduction and some expands on it. Consider merging and using the Introduction to frame the concept as a composite marker of cortical microstructure from the start.

In light of this review and our recent analyses on the reliability of intracortical T1w/T2w [(Paquola et al., 2025); https://doi.org/10.1101/2025.10.16.682836], we made two substantial revisions to the manuscript. First, we removed µ0 (mean intensity) from all analyses and now focus exclusively on µ1 (centre of gravity) and µ2 (variance). Second, we revised our terminology to describe these moments in relation to intracortical myelin rather than using the less precise term "microstructural density".

In the original submission we were cautious about framing T1w/T2w as a myelin marker, largely due to evidence that T1w/T2w does not correspond to inter-regional differences in myelin staining acquired in the same brain (Sandrone et al., 2023). However, our analyses of a multi-modal test-retest dataset (Shams et al., 2019) now show that individual-specific patterns of µ1 and µ2 derived from T1w/T2w and R1 are highly similar ($r = 0.95$) (Paquola et al., 2025). Given R1 is strongly correlated with histological myelin [$r = 0.805$ (Stuber et al., 2014)], this finding strengthens our confidence that these measures, derived from intracortical T1w/T2w, primarily reflect depth-wise variations in myelin. In contrast, µ0 shows substantially weaker correspondence between T1w/T2w and R1. This effect, as well as the limited histological validation reported by Sandrone et al. (2023), likely reflects B1+ transmit-field inhomogeneities, which are known to affect T1w/T2w intensities (Glasser et al., 2022). Higher-order moments (µ1 and µ2) are less susceptible to these biases because they encode relative intensity distributions within a region and are therefore more robust to low-spatial-frequence B1+ variations.

Following the Reviewer's recommendation, we have reframed our discussion of T1w/T2w in the Introduction, presenting it from the outset with regards to its sensitivity to intracortical myelin.

Introduction (P.5, L.66-81)

"Intracortical profiling of sub-millimetre resolution *in vivo* MRI offers a novel opportunity to tap into the micro-scale maturation of the cortex. Recent work has shown that microstructure profiles, extracted from T1-weighted/T2-weighted (T1w/T2w) imaging, capture regional differences in laminar organisation, as indicated by post-mortem histology (Paquola et al., 2025; Paquola et al., 2019b; Sui et al., 2022). T1w/T2w imaging enhances cortical myelin contrast by mathematically cancelling out receive field (B1-) bias (Glasser et al., 2014; Glasser & Van Essen, 2011; Nakamura et al., 2017). The residual transmit field (B1+) biases, however, vary across the field of view and across participants in such a way that absolute differences in T1w/T2w values cannot be directly interpreted as absolute differences in cortical myelin (Glasser et al., 2022; Sandrone et al., 2023). Inspecting relative T1w/T2w levels across cortical depths within an area overcomes this issue, however, due to the low spatial frequency of the B1+ biases (MacLennan et al., 2022) Indeed, using a multi-modal test-retest dataset (Shams et al., 2019), Paquola et al. (2025) recently showed that the shape of T1w/T2w intracortical profiles are reliable and exhibit very similar individual-specific spatial patterns to those derived from quantitative R1. Combined with evidence that R1 is strongly correlated with histological myelin (Stuber et al., 2014), these findings support that the intracortical balance of T1w/T2w effectively proxies depth-wise variations in myelin, and these values can be compared across participants."

R1#2: Sampling across 12 equivolumetric intracortical surfaces is interesting, but neonatal cortex is very thin (<2 mm, 3-4 voxels at 0.5 mm resolution). Surfaces are thus highly non-independent and susceptible to partial volume effects, interpolation errors, and noise. Regional differences in cortical thickness may also affect centre of gravity and variance estimates because of 'closer/wider' sampling, potentially impacting the resulting 'laminar' intensity distributions. It would be good to discuss this more explicitly.

We agree with the Reviewer that the thinness of the neonatal cortex limits the granularity at which intracortical profiling on in vivo MRI can resolve laminar organisation. Indeed, the 12-surface sampling scheme necessarily oversamples the underlying tissue, and adjacent surfaces are not statistically independent measurements. Our goal, however, is not to resolve individual cortical layers, which is not feasible at this resolution, but rather to capture the general *distribution* of the intracortical myelin. For this reason, we use central moments ($\mu 1$ and $\mu 2$) instead of depth-specific intensities. Central moments are less sensitive to the non-independence of neighbouring surfaces because they summarise the relative distribution of values across depth. In contrast, depth-wise intensity estimates are more directly influenced by surface spacing and partial volume fluctuations. As noted in Paquola et al. (2025), oversampling combined with trilinear interpolation increases the effective number of unique intensity samples and substantially reduces the dependence of the moments on cortical thickness—an issue that is more pronounced when using nearest-neighbour interpolation.

Further, recent work using ultra-high-resolution in vivo MRI (0.25 mm isotropic) shows that regional variation in $\mu 1$ and $\mu 2$ is highly preserved at lower resolutions (down to 1 mm) when using this oversampled, trilinearly interpolated approach ($r > 0.9$). Thus, although the present method cannot resolve discrete laminar boundaries, our previous analyses indicate that this sampling strategy is sufficient to recover reliable regional variation in terms of the broader laminar organisation of the cortex.

Regarding the specific concern about cortical thickness influencing the centre of gravity and variance, our sensitivity analyses support that this effect is minimal. Re-estimating all developmental models with cortical thickness included as a covariate yielded highly similar maps ($r > 0.93$ for both $\mu 1$ and $\mu 2$ across PMA, GA, and PNA). In addition, the use of equivolumetric surfaces ensures that sampling follows the local cortical geometry, reducing systematic morphological biases relative to equidistant spacing.

Together, these results suggest that while the surfaces are indeed non-independent, as expected given neonatal cortical thickness, the combination of (i) oversampling with trilinear interpolation, (ii) summarising profiles using central moments, and (iii) equivolumetric spacing provides a robust and thickness-insensitive characterisation of intracortical T1w/T2w distributions at the resolution available.

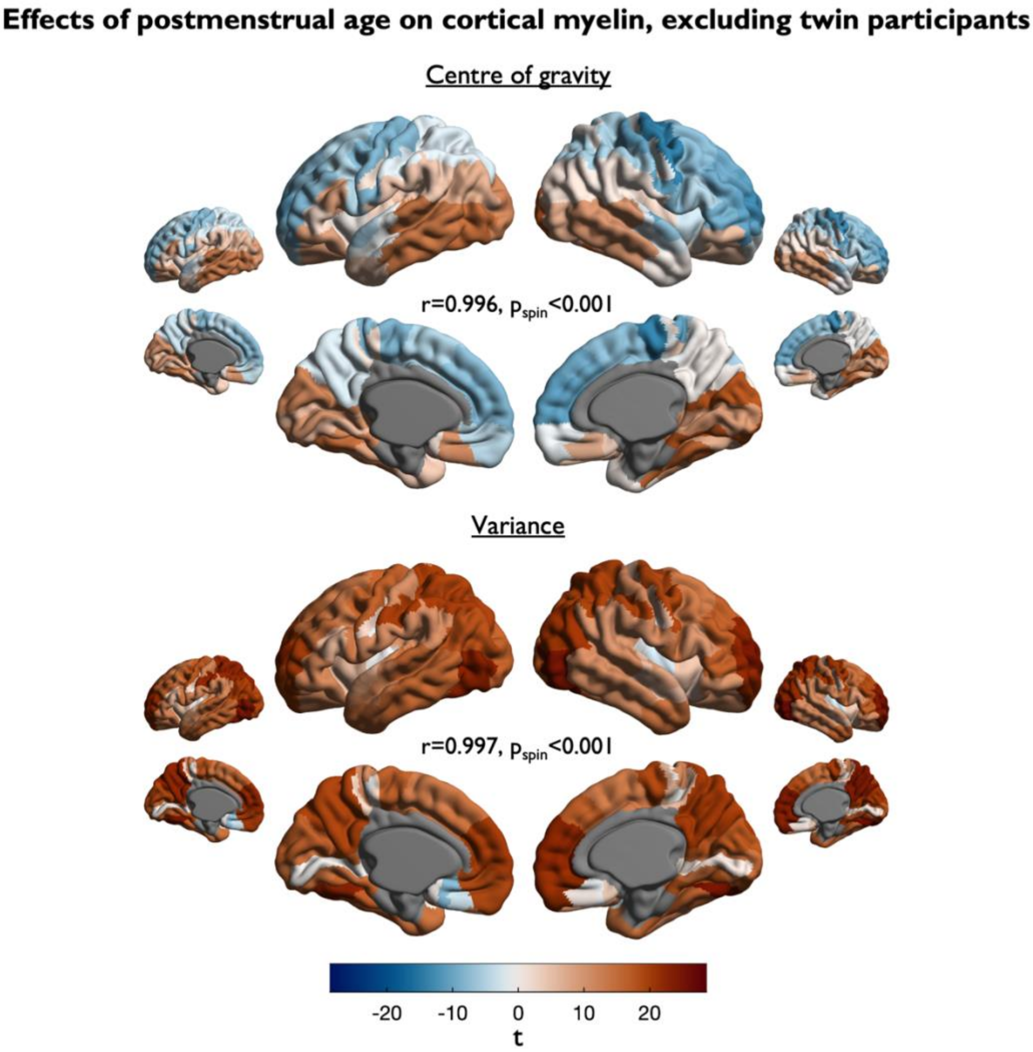

Supplementary figure 1: Linear regression models were used to assess the association between postmenstrual age (PMA) and intracortical profile moments across cortical regions, while controlling for sex. Bigger surface maps were derived from a subsample of participants, which excluded any participants of multiple pregnancies. Smaller surface maps were derived from the original sample of this study. All surface maps display t-values for the PMA-estimate, projected onto the cortical surface for centre of gravity (top) and variance (bottom). The spatial correlation between the effects on the original dataset and the dataset excluding twins is displayed in the middle of each set, together with the p-value derived from spin-based permutation testing (n = 10000), between the 2.5th to 97.5th percentile of the permuted correlations. Excluded parcels are displayed in grey.

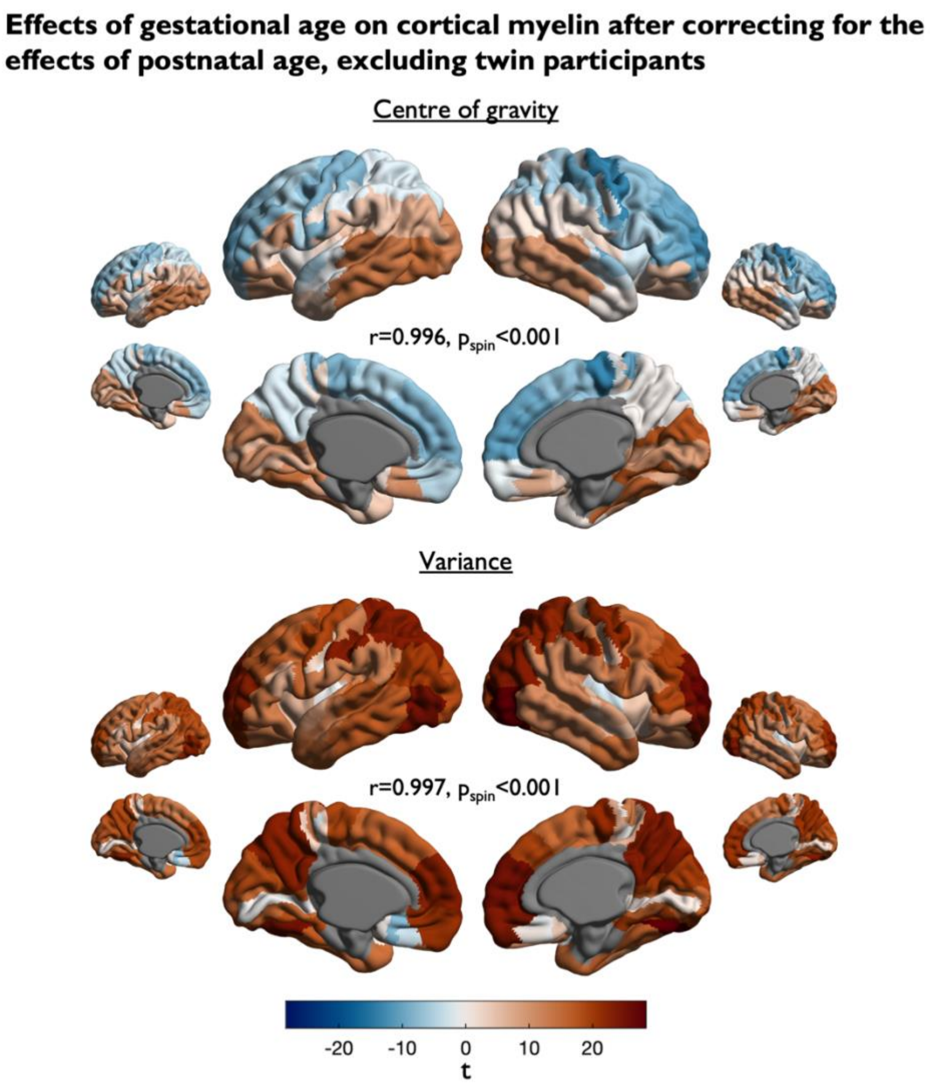

Supplementary figure 2: Linear regression models were used to assess the association between gestational age (GA) and intracortical profile moments across cortical regions, while controlling for sex. Bigger surface maps were derived from a subsample of participants, which excluded any participants of multiple pregnancies. Smaller surface maps were derived from the original sample of this study. All surface maps display t-values for the GA-estimate, projected onto the cortical surface for centre of gravity (top) and variance (bottom). The spatial correlation between the effects on the original dataset and the dataset excluding twins is displayed in the middle of each set, together with the p-value derived from spin-based permutation testing (n = 10000), between the 2.5th to 97.5th percentile of the permuted correlations. Excluded parcels are displayed in grey.

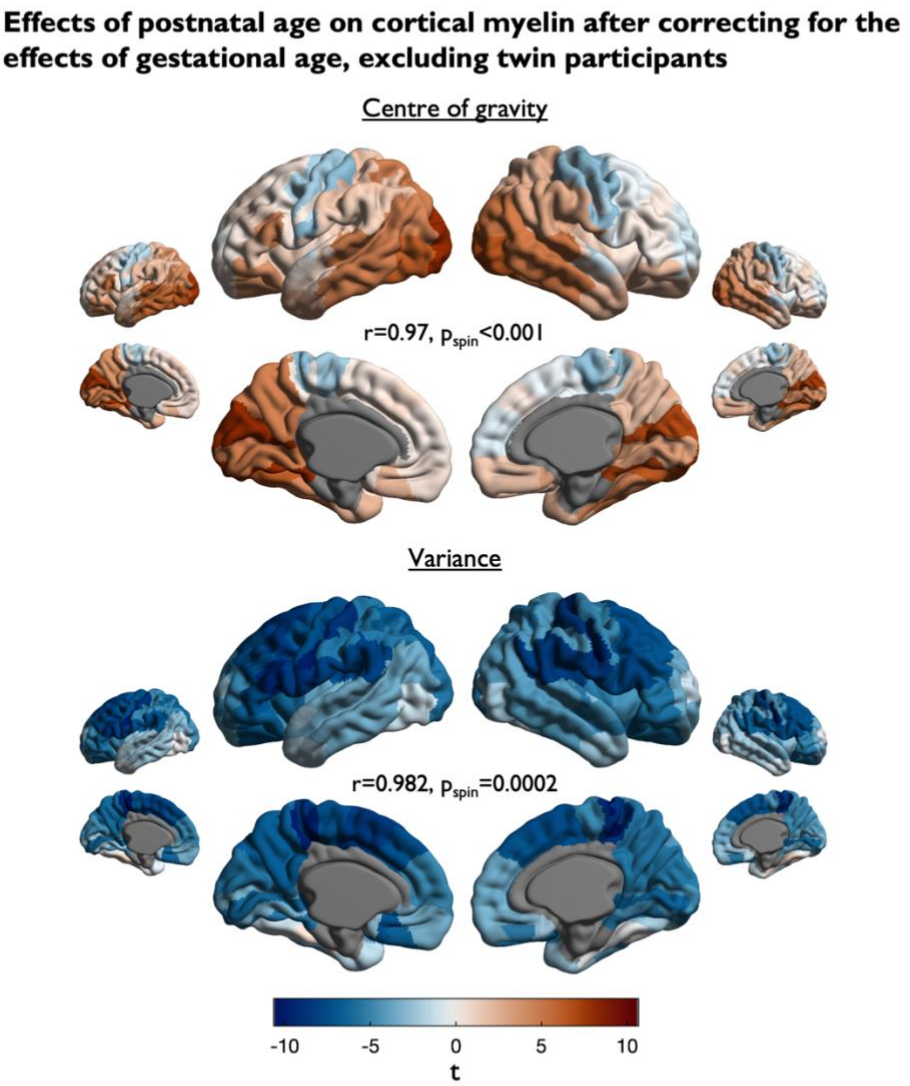

Supplementary figure 3: Linear regression models were used to assess the association between postnatal age (PNA) and intracortical profile moments across cortical regions, while controlling for sex. Bigger surface maps were derived from a subsample of participants, which excluded any participants of multiple pregnancies. Smaller surface maps were derived from the original sample of this study. All surface maps display t-values for the PNA-estimate, projected onto the cortical surface for centre of gravity (top) and variance (bottom). The spatial correlation between the effects on the original dataset and the dataset excluding twins is displayed in the middle of each set, together with the p-value derived from spin-based permutation testing (n = 10000), between the 2.5th to 97.5th percentile of the permuted correlations. Excluded parcels are displayed in grey.

Methods (P.15, L. 222-227)
"Together, the equivolumetric spacing, the oversampling using trilinear interpolation, as well as the use of central moments to summarise the intracortical profiles ensure a robust and thickness-insensitive characterisation of intracortical T1w/T2w distributions at the resolution available. This approach enables the reliable investigation of the regional variation of laminar organisation, somewhat overcoming the limitation of the relatively low resolution of the scans."

R1#3: Von Economo atlas is based on adult cytoarchitecture, raising concerns about the relevance of its regional boundaries in neonates, especially since your study focuses on laminar microstructure with goal to show that it significantly changes during the perinatal period (do you expect the laminae to change with age but the boundaries to stay exactly same as in adult?)
We agree that understanding how areal boundaries emerge and stabilise across the perinatal period is an important and fascinating question. Although this lies beyond the scope of the present study, we sought to ensure that our findings were not contingent on adult-derived cytoarchitectural boundaries.

To this end, we replicated all analyses using a finer-grained informed atlas (Schaefer-200). The spatial patterns were highly consistent across parcellations, indicating that our results are robust and not driven by the specific boundaries of the von Economo atlas.

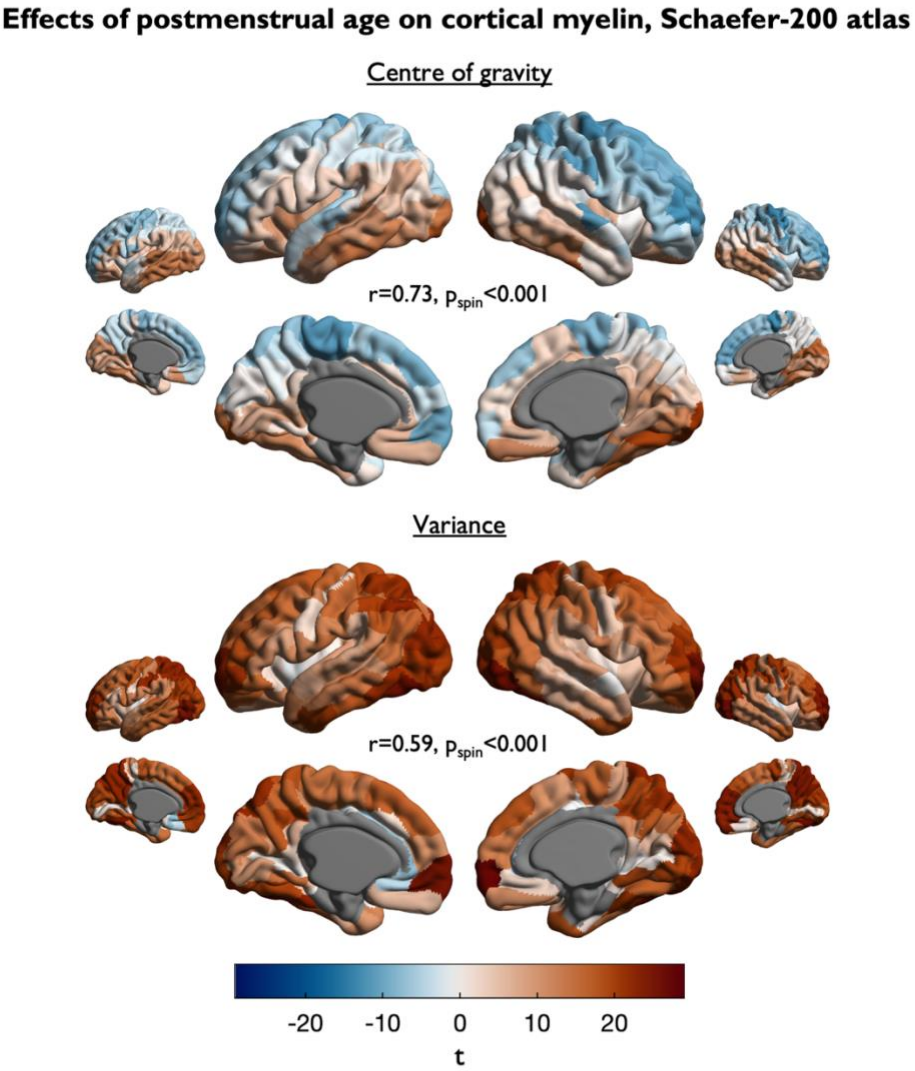

Supplementary figure 7: Linear regression models were used to assess the association between postmenstrual age (PMA) and intracortical profile moments across cortical regions of the Schaefer-200 atlas, while controlling for sex. Bigger surface maps display t-values for the PMA-estimate, projected onto the cortical surface for centre of gravity (top) and variance (bottom). Smaller surface maps show the results of the same models using the von Economo atlas. The spatial correlation between the effects on the Schaefer-200 and von Economo atlas is displayed in the middle of each set, together with the p-value derived from spin-based permutation testing (n = 10000), between the 2.5th to 97.5th percentile of the permuted correlations. To calculate these correlations, parcel-wise results were upsampled to vertex-wise level. Excluded parcels are displayed in grey.

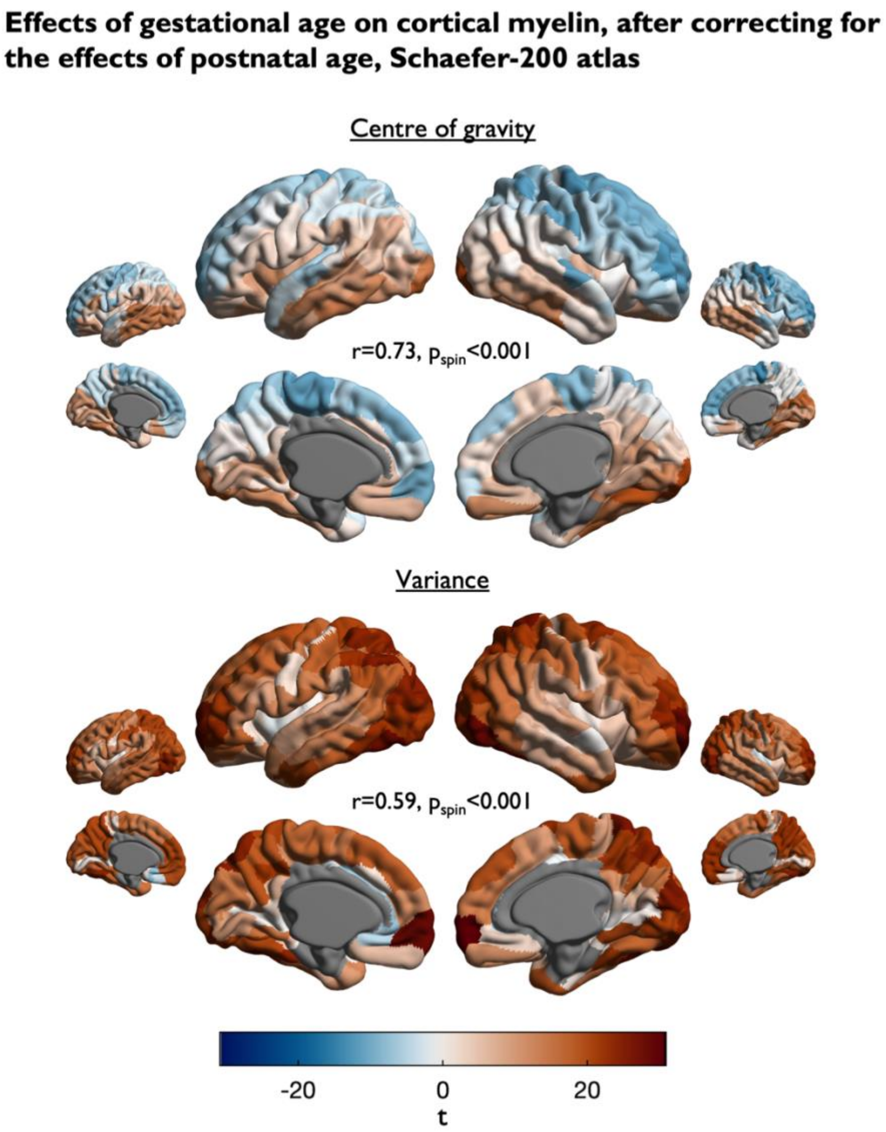

Supplementary figure 8: Linear regression models were used to assess the association between gestational age (GA) with intracortical profile moments across cortical regions of the Schaefer-200 atlas, while controlling for sex. Bigger surface maps display t-values for the GA-estimate, projected onto the cortical surface for centre of gravity (top) and variance (bottom). Smaller surface maps show the results of the same models using the von Economo atlas. The spatial correlation between the effects on the Schaefer-200 and von Economo atlas is displayed in the middle of each set, together with the p-value derived from spin-based permutation testing (n = 10000), between the 2.5th to 97.5th percentile of the permuted correlations. To calculate these correlations, parcel-wise results were upsampled to vertex-wise level. Excluded parcels are displayed in grey.

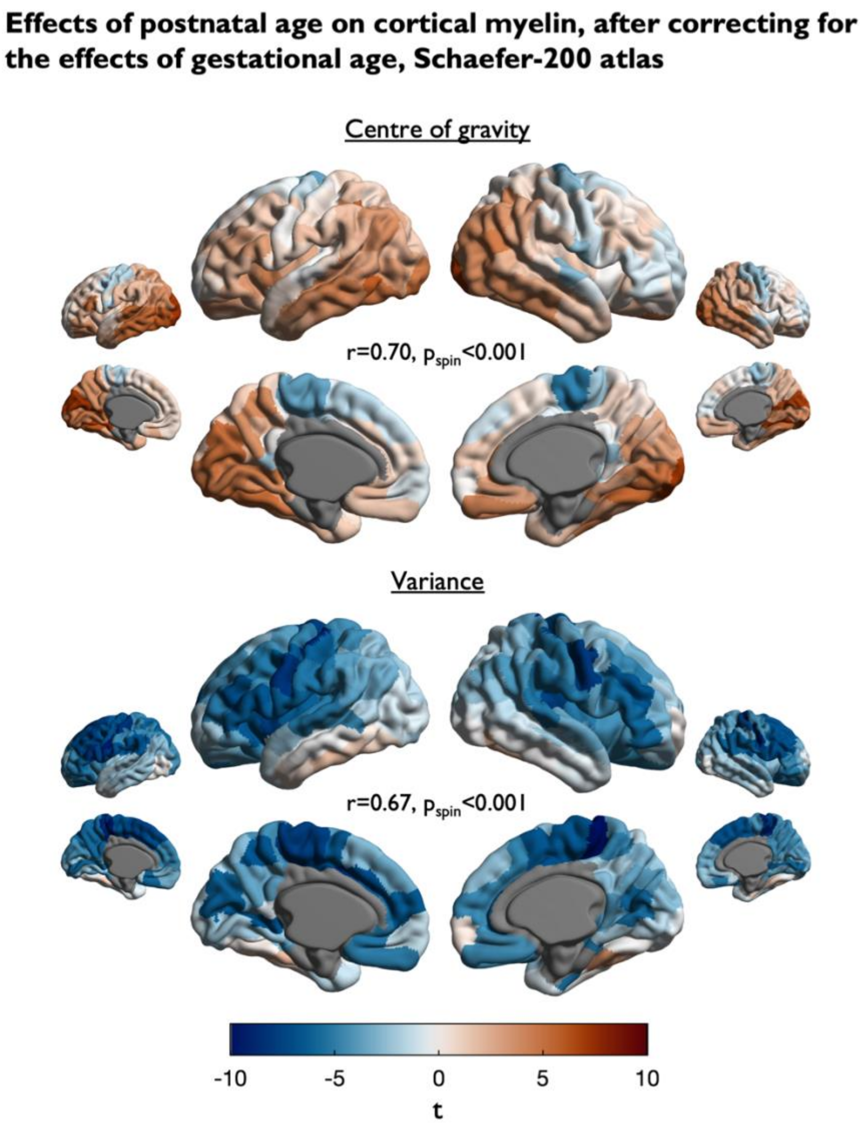

Supplementary figure 9: Linear regression models were used to assess the association between postnatal age (PNA) with intracortical profile moments across cortical regions of the Schaefer-200 atlas, while controlling for sex. Bigger surface maps display t-values for the PNA-estimate, projected onto the cortical surface for centre of gravity (top) and variance (bottom). Smaller surface maps show the results of the same models using the von Economo atlas. The spatial correlation between the effects on the Schaefer-200 and von Economo atlas is displayed in the middle of each set, together with the p-value derived from spin-based permutation testing (n = 10000), between the 2.5th to 97.5th percentile of the permuted correlations. To calculate these correlations, parcel-wise results were upsampled to vertex-wise level. Excluded parcels are displayed in grey.

R1#4: Generally, methodological limitations are not discussed in the Discussion at all; including these would better contextualize the results.

We thank the Reviewer for pointing this out. We now included a separate section on the limitations within the Discussion.

Discussion (P.26, L.410-424)
"This study was constrained by a number of limitations. First, the inclusion of extremely and very preterm infants makes it difficult to separate the influences of gestational timing from the impact of medical interventions that concern prematurely born infants (e.g. respiratory support, medication exposure, etc.). Although we performed sensitivity analyses excluding extremely and very preterm infants, residual confounding by these clinical factors cannot be excluded. Therefore, our assumptions regarding premature transition to the *ex utero* environment altering developmental trajectories of cortical growth must be interpreted as hypothetical interpretations and not as definitive conclusions.

Second, the cross-sectional design and narrow postnatal age range limit the potential to investigate developmental trajectories or to disentangle prenatal from postnatal influences. Gestational and postnatal age are collinear components of postmenstrual age and differ in their ranges, thus we can only infer relative associations with these developmental components, rather than strict separations of pre- vs postnatal influences. Finally, although our approach provides novel insights into the balance of myelin across cortical depths, the imaging resolution prohibits the delineation of separate cortical layers. As such, we may not make conclusions about the differentiation or development of numbered layers."

R1#5: 'Histological studies, that do directly assess cytoarchitectural features, are naturally cross-sectional and often very limited in sample size.' Your study is also cross-sectional 0:)
We thank the Reviewer for this clarification. While we agree that our cross-sectional study cannot fill this gap, our intention in this sentence was to highlight the potential of in vivo imaging to complement histological work in future longitudinal studies. However, in our revised manuscript this sentence has been removed.

R1#6: Framing of perinatal development.
The manuscript often presents perinatal development as a dichotomous shift from genetic to experience-driven processes. Ontogeny is continuous and shaped by complex interactions (maternal-fetal environment, epigenetics, metabolism, circuit maturation, sensory input). I recommend softening this framing and clarifying that observed changes reflect interacting processes rather than a strict intra- vs. extrauterine shift. I have also few hesitations about statements like:

- **'foetus is relatively isolated from sensory input' is not fully accurate. Fetal sensory input exists and differs from postnatal exposure.**
- **The claim that thalamocortical connectivity refines cortical arealisation postnatally is overly restrictive. Thalamocortical and corticocortical connections develop prenatally, with subplate activity already influencing laminar organization. Consider clarifying that these processes begin in utero and continue postnatally.**

We thank the Reviewer for this helpful feedback. We agree with their points and have rephrased the text accordingly.

Introduction (P.3, L.43-46)
"While *in utero* the foetus is sensitive to the maternal heartbeat, touch of the abdomen and external noise (Marx & Nagy, 2015; Webb et al., 2015), genetic programs primarily steer neurodevelopmental processes (Bystron et al., 2008; Webb et al., 2001)."

Discussion (P.25, L.393-395)
"Starting *in utero* and continuing during postnatal development, thalamocortical connectivity is thought to refine cortical arealisation, especially around primary sensory areas (Cadwell et al., 2019; Molnar & Kwan, 2024)."

R1#7: While trying to distinguish prenatal vs. postnatal effects is interesting, I'm not sure the data allow strong conclusions. You have access to scans at a specific age (PMA), and then you create prenatal and postnatal period as summative age birth and birth-scan (24wGA +6 weeks to scan does not represent the same developmental period as 38wGA + 6 weeks and correcting with linear regression is probably insufficient to disentangle these differences).

Also, GA and PNA differ in duration (months vs. max 7 weeks), so caution is warranted when interpreting relative effects (for example saying prenatal development has larger effects).
We thank the Reviewer for raising this important point. We fully agree that GA and PNA are not independent of PMA (GA+PNA=PMA), therefore it is not possible to completely separate prenatal and postnatal influences. Our simultaneous modelling of GA and PNA therefore addresses a more limited question of whether infants who spent relatively more time in utero vs ex utero show systematic differences in intracortical microstructure. Therefore, the analysis aims at disentangling GA and PNA

as distinct associations with cortical microstructure conditional on PMA, but not at reconstructing the true underlying developmental trajectories. As the Reviewer notes, infants with the same PMA but different GA (e.g. 24+6 vs. 38+6 weeks) may follow different biological paths that a linear model cannot fully capture.

In the revised manuscript, we explicitly note that because GA and PNA differ in duration, we interpret their effects as relative associations rather than directly comparable magnitudes.

Methods (P.8, L.111-115)
"Since gestational and postnatal age are not independent of postmenstrual age, it is not possible to biologically separate the influences of each period. However, we aimed to partially disentangle their distinct associations with cortical microstructure conditional on postmenstrual age, in lieu of being able to reconstruct individualised developmental trajectories throughout the *in- and ex-utero* periods."

Methods (P.17, L.241-246)
"Due to the broad overlap of the distributions of gestational and postnatal age, we isolated the unique contributions of each developmental variable to cortical microstructure in multi-variate regression models, that included gestational age, postnatal age and sex. Since gestational and postnatal age are not independent from postmenstrual age, we used the multi-variate models to assess the effects of these developmental metrics as relative associations and not to directly compare their magnitudes."

Results (P.20, L.286-292)
"To examine how the timing of birth and the developmental period after birth influence early cortical development, we linearly modelled the relationship of each central moment with gestational age and postnatal age, while controlling for the effect of the other (**Figure 4**). This approach doesn't aim at completely separating prenatal and postnatal influences, but rather to investigate the systematic differences in intracortical microstructure between infants who spent relatively more time *in utero* and infants who spent more time *ex utero*."

Discussion (P.26, L.418-421)
"Gestational and postnatal age are collinear components of postmenstrual age and differ in their ranges, thus we can only infer relative associations with these developmental components, rather than strict separations of pre- vs postnatal influences."

R1#8: Observed laminar differences (sensorimotor deeper vs. visual upper layers) are intriguing. You link this to later functional differentiation in discussion. Would this potentially reflect developmental asynchronies; sensorimotor areas might mature earlier, and superficial-to-deep maturation may place them in a 'deeper stage' at the observed ages (tentative).

We appreciate this thoughtful comment and agree that regional developmental asynchronies provide a plausible explanation for the laminar differences we report. While revising this section of our Discussion, we noticed an error in the text, where the direction of the described effects was accidentally swapped between the sensorimotor and visual areas. As shown in our effect maps, the PNA-related increases of T1w/T2w in visual areas are stronger in the deeper cortical layers, whereas in sensorimotor areas increases occur in superficial layers. The changes in visual areas could possibly reflect myelination of deep-layer axons and changes in sensorimotor areas could be related to greater increases in upper-layer intensities and/or smaller changes in deeper layers. However, although our analysis can discriminate depth-dependent microstructural changes between sensorimotor and visual areas, it does not allow us to temporally differentiate their maturation. Even considering a superficial-to-deep or inside-out maturation, our metrics describe the distribution of T1w/T2w intensities across cortical depths and are agnostic to underlying cytoarchitectonic layers or the timing of neuronal migration. Thus, our data can't determine whether sensorimotor regions are "more advanced" than visual areas in a developmental sense. We have included this in the Discussion section of our revised manuscript.

Discussion (P.25, L.397-408)

"While the short postnatal timeframe encompassed by this dataset precludes interpretation of maturity per se, we observed strong postnatal changes in deeper layers of the occipital and inferior temporal cortex, highlighting a preferential development of deeper layers of cortical regions involved in visual processing during this early period of immense sensory stimulation. Notably, we found an opposing trend in sensorimotor areas, with a preference towards microstructural development of upper layers. This contrast may underpin the differentiation of sensorimotor and visual cortical areas; a pattern that aligns with their strong functional differentiation during infancy (Lariviere et al., 2020). However, although intracortical profiling allows the characterisation of depth-dependent differences between regions, central moment metrics are agnostic to the timing of neuronal migration and layer formation, therefore it's not possible to determine whether one of these regions is "more advanced" than the other in a developmental sense."

R1#9: Handling of preterm and non-singleton infants

'This modulatory transition highlights the importance of the timing of birth in brain development, with prematurely born infants being exposed to a sensory-rich environment before the scaffold of their circuitry has been completely established for postnatal plasticity.' Exactly, the premature babies should not be considered as developing 'typically' so mixing them into your population might be problematic. 30% of the sample is preterm, which complicates interpretation of GA vs. PNA effects. This might need to be explicitly acknowledged.

We thank the Reviewer for raising this point. We should first clarify that we don't aim to model a typically developing population, but rather to disentangle the relative contributions of GA and PNA. To achieve sufficient variation in both components, we need a wide range of both pre- and postnatal developmental periods, including those of prematurely born individuals. If we restrict our analysis to full-term infants, the variance in GA will be restricted significantly, preventing us from estimating the independent association of GA with intracortical microstructure. We agree however, that including extremely preterm infants introduces additional complexity in the biological interpretation of our results, as their cortical microstructure is influenced not only by the timing of birth, but also by other clinical factors, such as respiratory support, medication exposure, etc. Our models cannot fully disentangle these influences, therefore we now specifically address this limitation in the manuscript. We have also performed additional sensitivity analyses excluding very and extremely preterm infants.

Methods (P.9, L.126-130)

"The inclusion of preterm infants in the study aims at providing a wide range of both pre- and postnatal developmental periods to be able to estimate the independent association of gestational age with intracortical microstructure as well. Results were replicated using the same sample of participants but excluding the participants of non-singleton pregnancies (**Supp. Fig. 1-3**), as well as excluding extremely and very preterm infants (**Supp. Fig. 4-6**)."

Discussion (P.26, L.410-414)

"First, the inclusion of extremely and very preterm infants makes it difficult to separate the influences of gestational timing from the impact of medical interventions that concern prematurely born infants (e.g. respiratory support, medication exposure, etc.). Although we performed sensitivity analyses excluding extremely and very preterm infants, residual confounding by these clinical factors cannot be excluded."

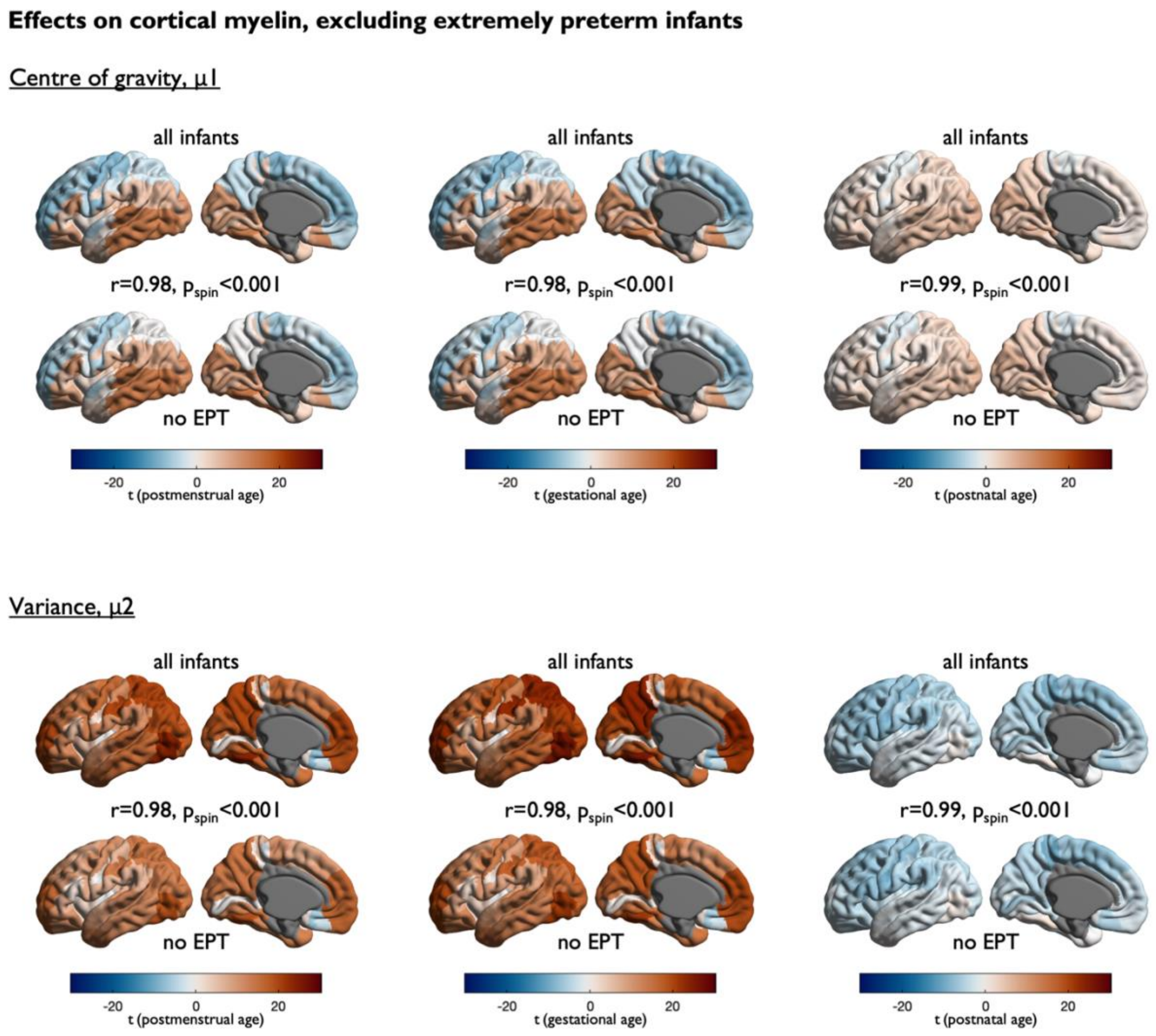

Supplementary figure 4: Linear regression models were used to asses the association between postmenstrual (left), gestational (middle) and postnatal age (right) and intracortical profile moments across cortical regions, while excluding extremely preterm (EPT) infants from the analyses. The first and third rows of surface maps display the t-values for the developmental variables in the original dataset with all participants, whereas the second and forth rows display the t-values for the developmental variables in the subset excluding EPT participants. The first two rows correspond to the linear models with centre of gravity as response variable, whereas the third and forth rows correspond to the models with variance as response variable. Excluded parcels are displayed in grey.

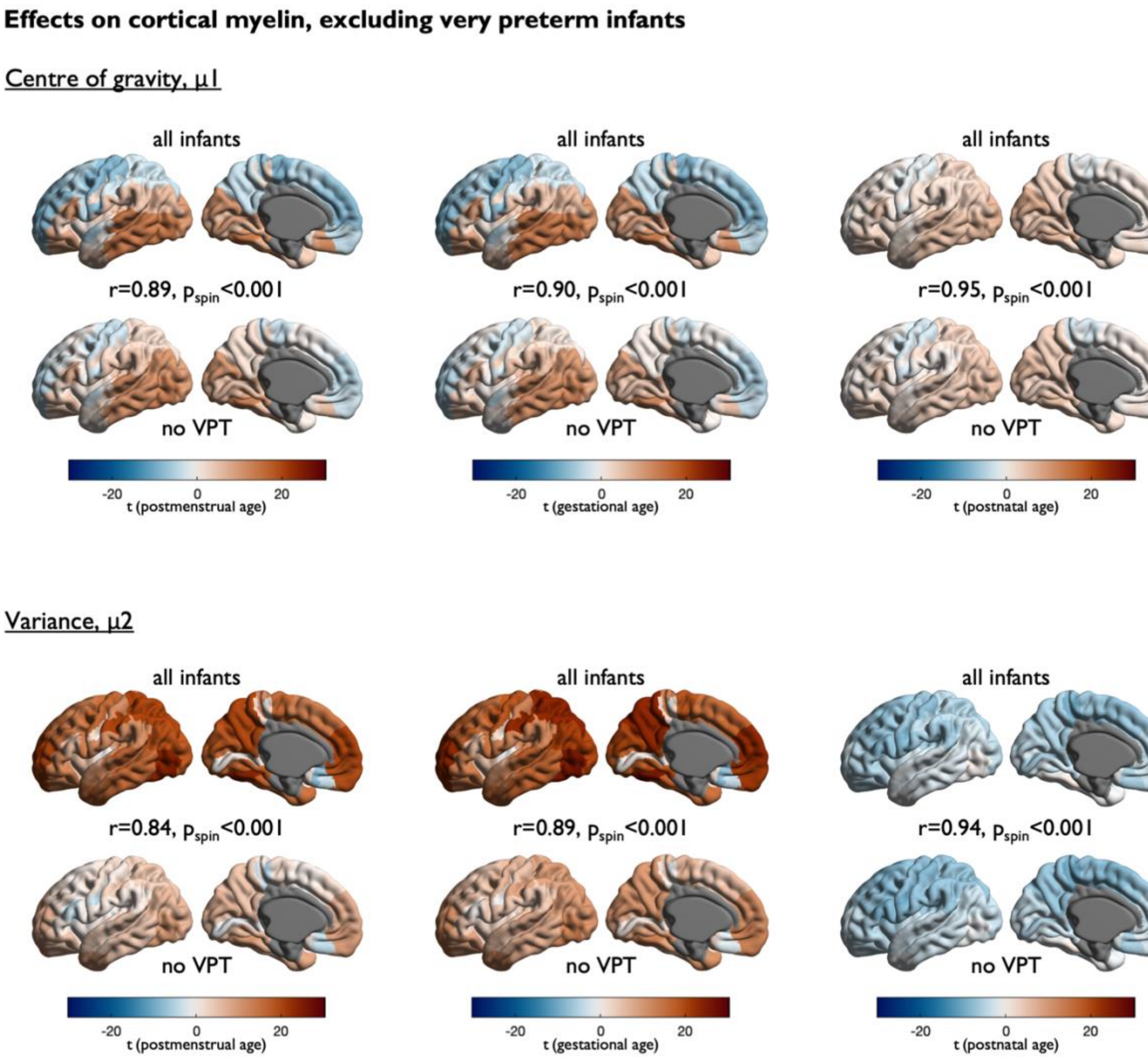

Supplementary figure 5: Linear regression models were used to asses the association between postmenstrual (left), gestational (middle) and postnatal age (right) and intracortical profile moments across cortical regions, while excluding very preterm (VPT) infants from the analyses. The first and third rows of surface maps display the t-values for the developmental variables in the original dataset with all participants, whereas the second and forth rows display the t-values for the developmental variables in the subset excluding VPT participants. The first two rows correspond to the linear models with centre of gravity as response variable, whereas the third and forth rows correspond to the models with variance as response variable. Excluded parcels are displayed in grey.

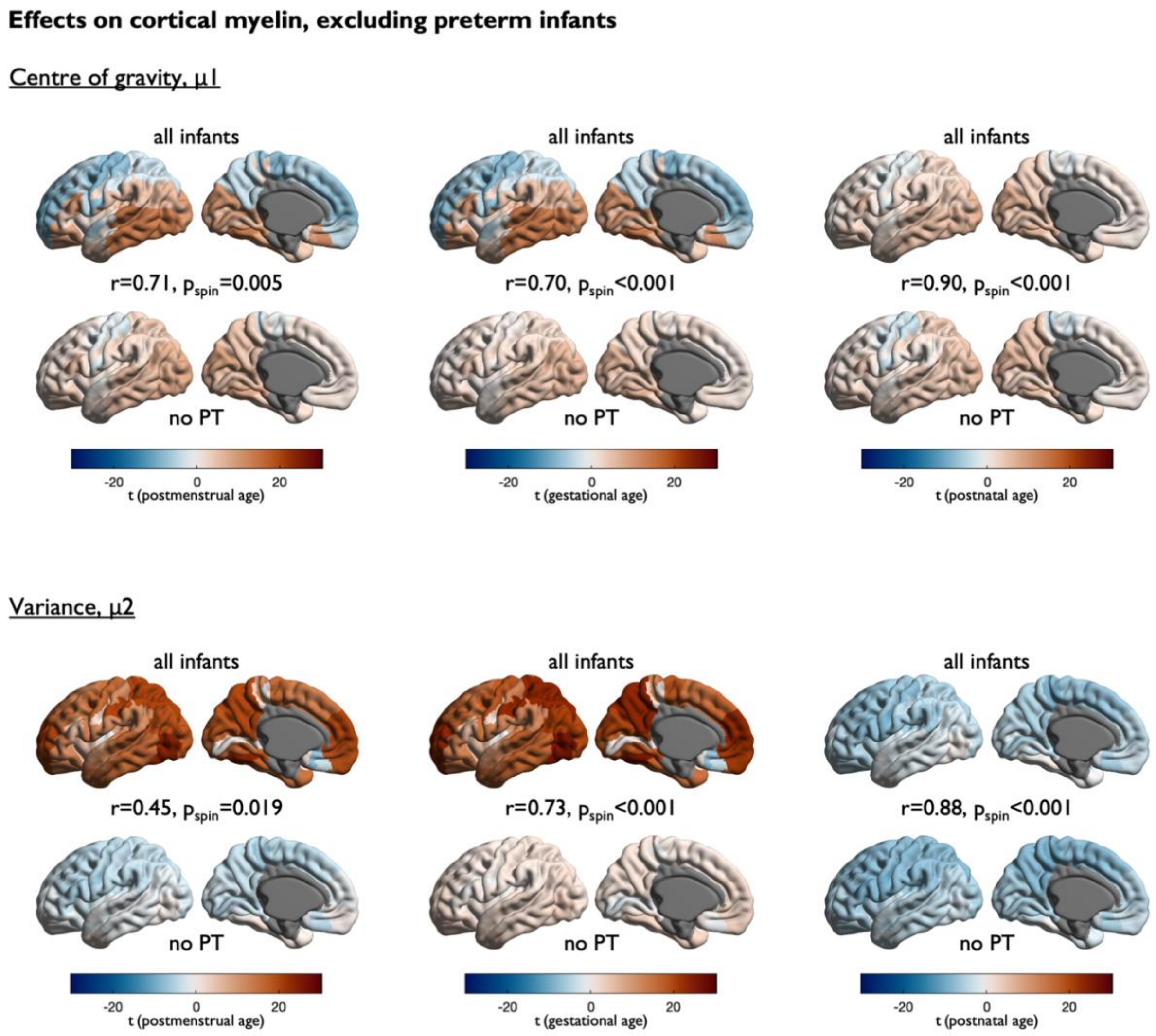

Supplementary figure 6: Linear regression models were used to asses the association between postmenstrual (left), gestational (middle) and postnatal age (right) and intracortical profile moments across cortical regions, while excluding preterm (PT) infants from the analyses. The first and third rows of surface maps display the t-values for the developmental variables in the original dataset with all participants, whereas the second and forth rows display the t-values for the developmental variables in the subset excluding PT participants. The first two rows correspond to the linear models with centre of gravity as response variable, whereas the third and forth rows correspond to the models with variance as response variable. Excluded parcels are displayed in grey.

R1#10: The rationale for excluding scans with PNA >7 weeks is unclear. You assume equivalent postnatal weeks after preterm vs. term birth reflects the same developmental stage, it might be clarified bit more.

We thank the Reviewer for this suggestion. We have adapted the corresponding text in the Methods. In short, infants who were scanned that late after birth were exclusively preterm infants (13 extremely preterm, 4 very preterm and 9 preterm), which likely due to medical complications couldn't be scanned closer to birth. Therefore, the threshold of PNA<7 weeks didn't only cover the mismatch in PNA distributions between preterm and full-term participants but also worked as an exclusion criterion for preterm participants with considerable medical complications.

Methods (P.9, L.121-124)

"Finally, since the oldest term-born infant had a postnatal age of 6^{+6} weeks, scans of preterm neonates with a postnatal age exceeding 7 weeks were also excluded to cover the mismatch in postnatal age distributions. This also worked as an exclusion criterion for preterm participants which were scanned significantly later after birth due to medical complications."

R1#11: 'In general, the prominent effect of gestational maturation on cortical microstructure emphasises the importance of the timing of birth and indicates that premature transition to the ex utero environment could disturb the well-regulated developmental trajectories of cortical growth, an insight that aligns with literature across various modalities' I don't disagree with the statement, but you did not study this directly?

We agree with the Reviewer and have revised the manuscript to clarify that our findings are consistent with prior literature that has suggested this.

Discussion (P.24, L.375-379 … 381-384)

"In general, the prominent effect of gestational maturation on cortical myelin hints toward the importance of the timing of birth and is consistent with literature across various modalities supporting that premature transition to the *ex utero* environment may alter the otherwise well-regulated developmental trajectories of cortical growth (Ball et al., 2013; Dimitrova et al., 2021; Fenn-Moltu et al., 2023; Franca et al., 2024)." … "Thus, the idea that earlier transition to the *ex utero* environment influences the trajectories of postnatal cortical development should be viewed as a speculative interpretation, rather than a conclusion from our analyses."

R1#12: Non-singleton participants (79 infants) are referred to as twins in supplementary materials. Why is the number not even, or maybe there were >2 non-singletons? Results on singletons are shown in supplementary, a brief mention in the main text, noting agreement with full sample, would strengthen the results. Otherwise one could argue that non-singletons might not be developing typically due to very different intra-uterine conditions compared to singletons, affecting your developmental relationships. (Similarly, it is very good that you performed the same analysis with the Schaefer atlas too, why are the results not ever mentioned/discussed? Even 1-2 sentences about whether the results agree or not, and why would be helpful.)

The number of non-singleton participants is not even because in some of the multiple births, only one of the twins met the inclusion criteria, whereas the other twin did not and was therefore excluded from the study. In such cases we kept the scan of the eligible infant in the analysis in order to preserve the statistical power, while still including them as non-singleton in the count of non-singletons, as they were born under shared intra-uterine conditions. The original dataset also includes >2 non-singletons (i.e. triplets), but none of them met the inclusion criteria of our study.

We thank the reviewer for the suggestion of referring to the sensitivity analyses without non-singletons and using the Schaefer atlas. We have adjusted our text accordingly.

Results (P.19, L.297-300)

"These effects were relatively consistent with a different parcellation scheme ($r > 0.7$ for centre of gravity and $r > 0.59$ for variance; **Supp. Fig. 7-9**) and when excluding extremely preterm ($r > 0.98$; **Supp. Fig. 4**), very preterm ($r > 0.89$; **Supp. Fig. 5**) and preterm infants ($r > 0.70$; **Supp. Fig. 6**)."

R1#13: Not sure I agree with the statement suggesting longitudinal studies could 'compensate for the imbalance in postnatal ages'. Longitudinal studies allow observation of trajectories, not correction of GA/PNA differences, or maybe you meant something else?

We thank the reviewer for this clarification and agree that longitudinal studies would not completely overcome the differences in GA and PNA. In the revised manuscript, we have softened this statement and we now emphasise that longitudinal studies would complement cross-sectional approaches by additionally enabling the observation of within-subject developmental trajectories.

Discussion (P.24, L.384-387)

"Longitudinal studies extending into later infancy would be especially helpful for elucidating how and when the modest postnatal age influences observed here evolve into larger, experience-driven changes. Such work could complement cross-sectional approaches by providing within-subject developmental trajectories."

R1#14: Consider reporting regional variability in intensity distributions across cortical depth as this is interesting in itself.

As discussed in R1#2, the depth-wise intensities are highly collinear. Nevertheless, we present a visualisation of depth-wise intensities in a new Supplementary Figure, as well as summary statistics of their mean and standard deviation per depth.

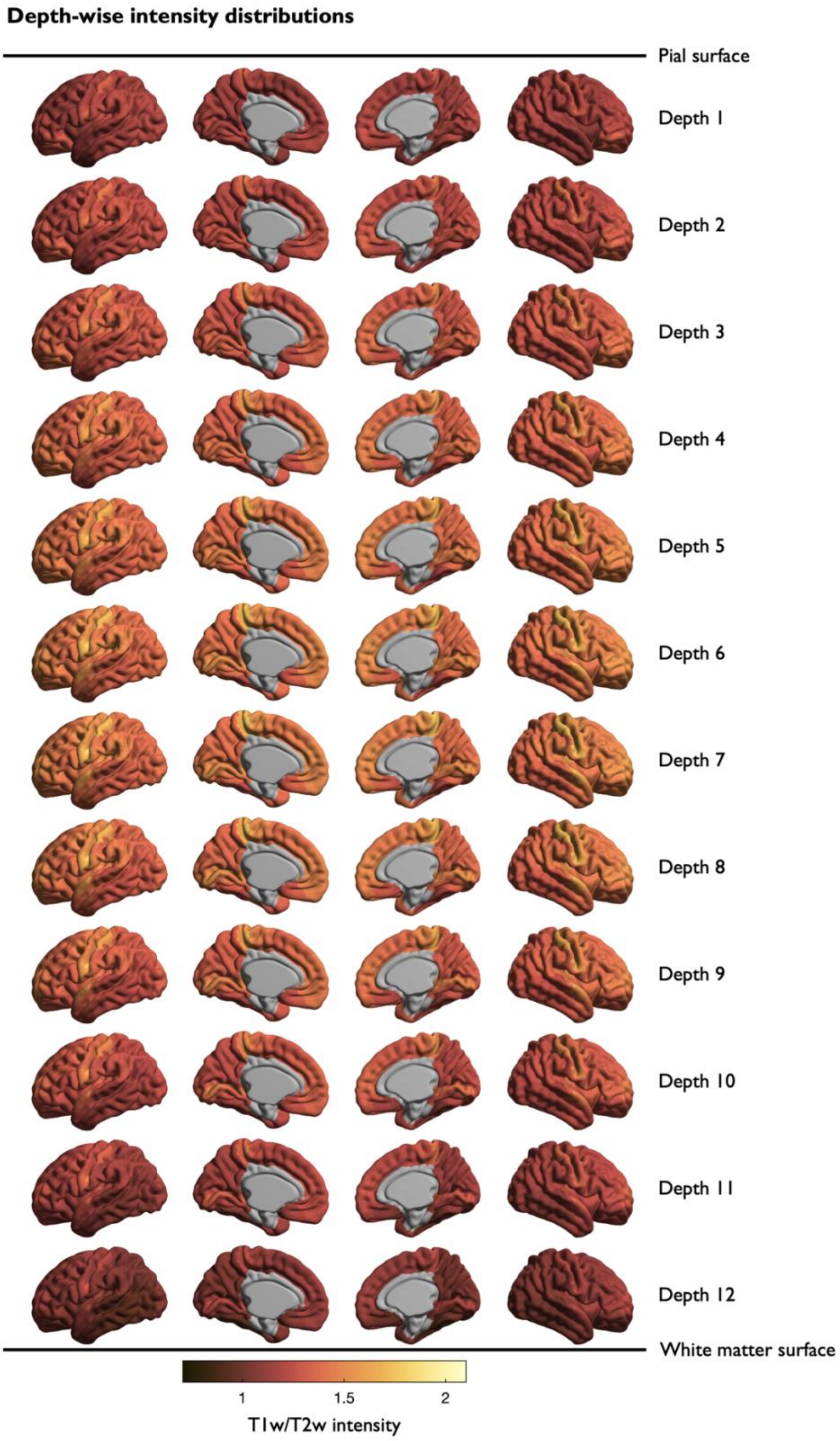

Supplementary figure 10: Parcel-wise T1w/T2w intensity distributions mapped on the dHCP 40-week surface template. Excluded regions (i.e. von Economo areas L_{A1}, L_{A2}, L_{C1}, L_{C2}, L_{C3}, L_D and L_E and the cortical wall) are shown in grey.

Table 1: Mean T1w/T2w intensities and standard deviations at each cortical depth for all infants, preterm and full-term infants. Depth 1 is closest to the pial surface, whereas depth 12 is closest to the white matter surface.

	All infants	Preterm infants	Full-term infants
**Depth 1**	1.20 ± 0.46	1.01 ± 0.38	1.27 ± 0.47
**Depth 2**	1.26 ± 0.46	1.07 ± 0.38	1.33 ± 0.47
**Depth 3**	1.32 ± 0.46	1.13 ± 0.38	1.39 ± 0.47
**Depth 4**	1.37 ± 0.47	1.18 ± 0.38	1.44 ± 0.47
**Depth 5**	1.40 ± 0.47	1.21 ± 0.38	1.47 ± 0.48
**Depth 6**	1.42 ± 0.48	1.23 ± 0.39	1.49 ± 0.49
**Depth 7**	1.42 ± 0.48	1.23 ± 0.39	1.49 ± 0.49
**Depth 8**	1.39 ± 0.48	1.21 ± 0.39	1.46 ± 0.49
**Depth 9**	1.35 ± 0.47	1.16 ± 0.39	1.42 ± 0.48
**Depth 10**	1.28 ± 0.47	1.10 ± 0.38	1.35 ± 0.48
**Depth 11**	1.20 ± 0.46	1.02 ± 0.38	1.27 ± 0.47
**Depth 12**	1.12 ± 0.46	0.94 ± 0.37	1.19 ± 0.47

R1#15: It would be nice to get more details on how the intensity values were sampled for the 12 intracortical surfaces (including interpolation).

We clarified the method of intracortical in more detail in the Methods of our manuscript.

Methods (P.13, L.182-184)

"Then, we sampled image intensities at matched vertices across the 12 intracortical depths using trilinear interpolation, which calculates a weighted average of eight neighbouring voxels at each sampling depth (**Figure 2A**)."

R1#16: Why are results in the main text shown for only one hemisphere? Were left/right regions averaged? Supplementary figures show both.

We didn't average across hemispheres, but since the effects look very similar in both hemispheres (inter-hemispheric correlation of effects >0.8 for centre of gravity and >0.9 for variance), we decided to show only the left one in the main figures for simplicity.

Figure 3 caption

"Due to the high inter-hemispheric correlation of the PMA-effects (r = 0.84 for centre of gravity and r = 0.96 for variance), we show only results of the left hemisphere for simplicity."

Figure 4 caption

"Due to the high inter-hemispheric correlation of the GA- and PNA-effects (r_{GA} = 0.84 and r_{PNA} = 0.8 for centre of gravity; r_{GA} = 0.96 and r_{PNA} = 0.9 for variance), we show only results of the left hemisphere for simplicity."

R1#17: The legend to Fig. 2 may not need to re-explain definitions of the signal descriptors, you provided those in the methods.

We thank the Reviewer for this suggestion. We have removed the redundant text from the caption.

Figure 2 caption

"**A)** Intracortical intensity profiles were extracted at each vertex of infant structural MRI scans. Intensities were sampled at twelve equivolumetric intracortical surfaces, spanning from the pial boundary (blue) to the white matter boundary (yellow), capturing signal variations across cortical depths, defined as microstructure profiles. **B)** The shape of the average microstructure profile within each von Economo parcel was summarised using two central moments: centre of gravity, and variance. **C)** Parcel-wise central moment distributions mapped on the dHCP 40-week surface template. Excluded

regions (i.e. von Economo areas L_{A1}, L_{A2}, L_{C1}, L_{C2}, L_{C3}, L_D and L_E and the cortical wall) are shown in grey. Due to the high inter-hemispheric correlation of the central moments (r = 0.79 for centre of gravity and r = 0.93 for variance), we show only results of the left hemisphere for simplicity."

R1#18: I find it strange that y-axis of the T1w/T2w intensity plots have no indication of 'localisation' even just adding pial,wm would be useful.

We appreciate the Reviewer for pointing this out. The axis legends of the intensity plots were adjusted accordingly. Changes in Figure 2 can be seen below and changes in Figure 3 can be found in comment #19, where the figure is attached.

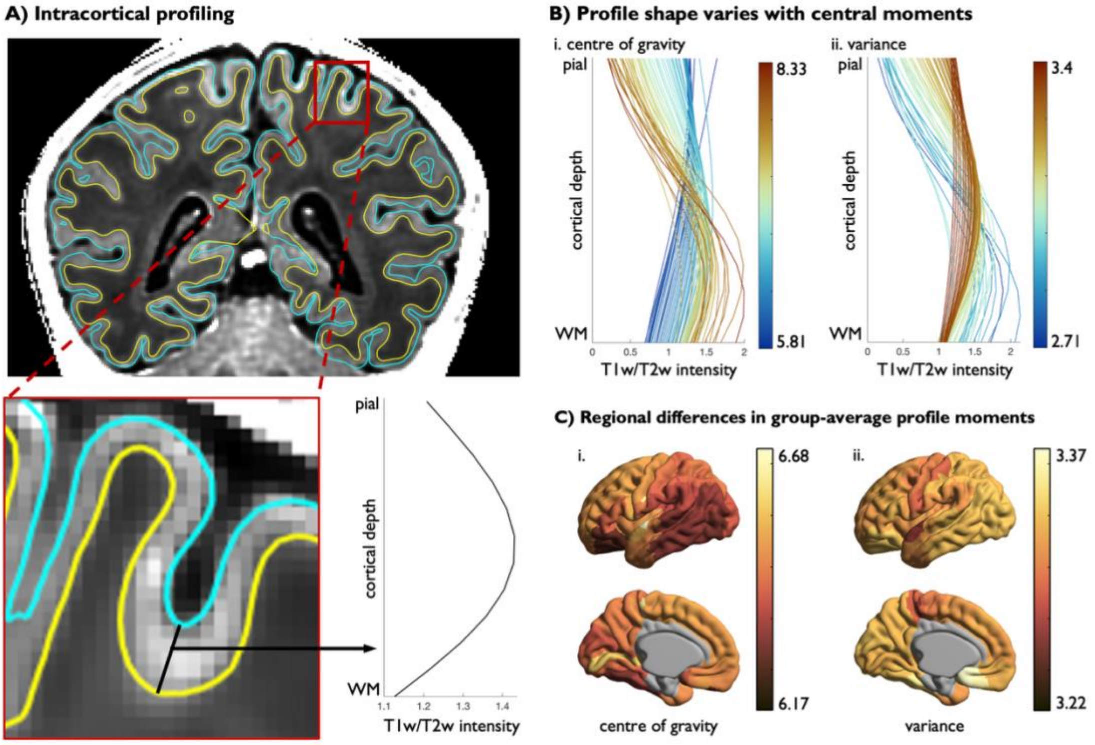

Figure 4: A) Intracortical intensity profiles...

R1#19: Figure 3: Why were correlations with variance not shown?

We appreciate this question from the Reviewer. We have adjusted Figure 3, showing the profile changes for the regions with the two highest increases/decreases in centre of gravity and variance.

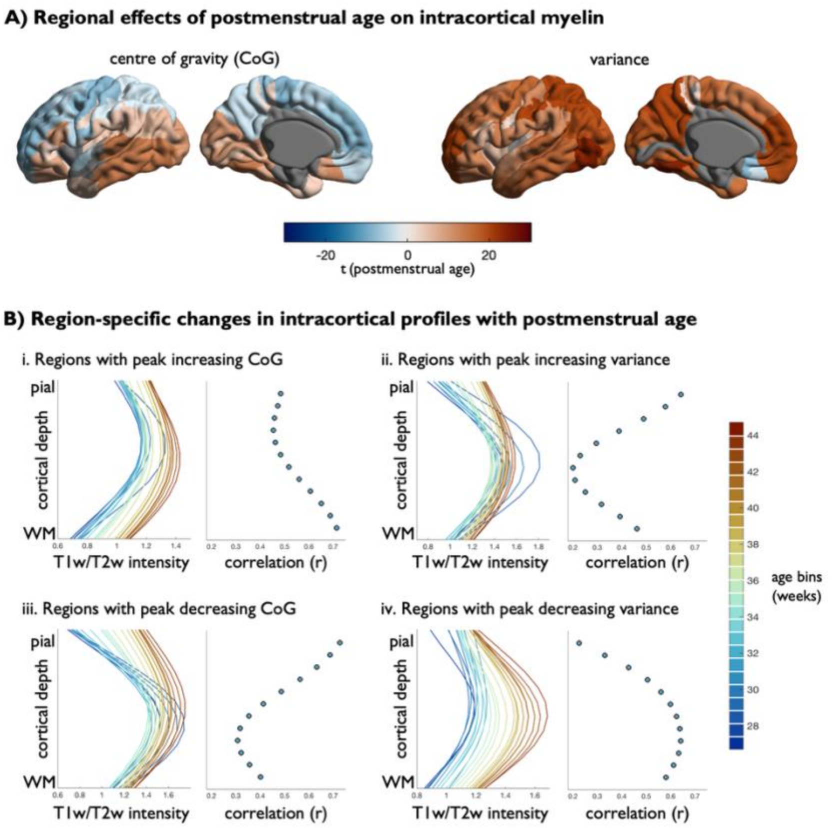

Figure 3: A) Linear regression models were used to assess the association between postmenstrual age (PMA) and intracortical profile moments across cortical regions, while controlling for sex. Surface maps display t-values for the PMA-estimate, projected onto the cortical surface for centre of gravity (left) and variance (right). Statistically non-significant parcels ($p > 0.05$) and excluded parcels are displayed in grey. **B)** Sliding window visualisation of profile changes with PMA (age bins = 1.5-week windows with 50% overlap) for the two regions with the maximum increases and decreases for centre of gravity (panel i and iii) and variance (ii and iv), with neighbouring scatter plots depicting the relationship between PMA with depth-specific T1w/T2w-intensities based on product-moment correlations. Due to the high inter-hemispheric correlation of the PMA-effects ($r = 0.84$ for centre of gravity and $r = 0.96$ for variance), we show only results of the left hemisphere for simplicity. Abbreviations: CoG: centre of gravity; PMA: postmenstrual age.

R1#20: Figure 5A: top right + bottom left and B left + middle - are these exactly same maps and correlations or are they so similar?

To clarify, these are the maps that are visible in Figure 3A and the top row of Figure 4, but without statistical significance threshold (i.e. all effects regardless of statistical significance). As pointed out in the Results and Discussion, these maps are very highly correlated, hence why they appear identical.

Figure 5 caption

"**A)** Surface maps of the first four vertex-wise geometric eigenmodes **B)** Product-moment correlation coefficients between the effects of postmenstrual (left), gestational (middle) and postnatal age (right) on each central moment and the 2nd-4th eigenmodes. The surface maps shown below each scatter plot are the same maps shown in Figures 3 and 4, but without significance threshold. The whiskers represent the range of correlation values obtained from spin-based permutation testing ($n = 10000$), between the 2.5th to 97.5th percentile of the permuted correlations. Significant correlations are highlight with one asterisk for $p < 0.05$ and two asterisks for $p < 0.01$. **B)** Linear regression models were used to assess the explained variance of the effects of postmenstrual (left), gestational (middle) and postnatal age (right) by the 2nd-4th geometric eigenmodes or combinations thereof."

R1#21: Several figure labels are inconsistent, e.g., "duration of gestation" in Figure 1B is Gestational age? Please double check the terminologies for ages (GA, PMA, PNA) throughout the manuscript and figures.

We thank the reviewer for point noticing this. We have adjusted the figure labels appropriately.

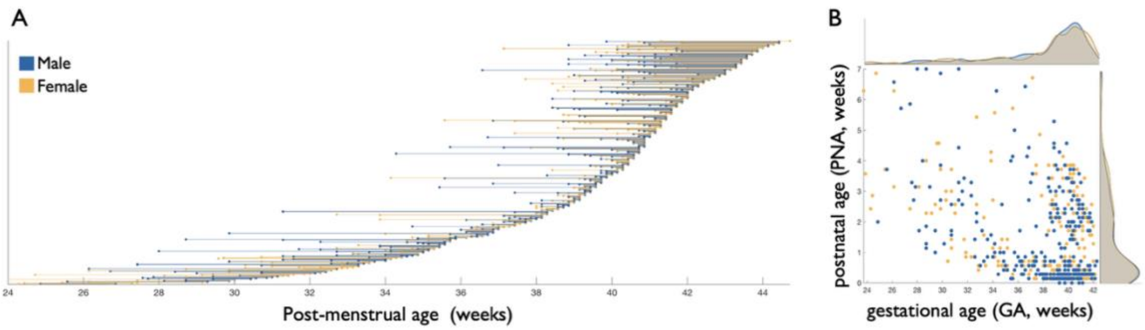

Figure 5: A) Age distributions of the 599 participants (y-axis). The plot represents duration of gestation at time of birth (left dot) and age at time of scan (right dot). **B)** The scatter plot illustrates the relationship between gestational age (GA) and postnatal age (PNA), with data points colour-coded by sex (blue: male, yellow: female). Histograms on the edges of the scatter plot show the distributions of gestational (top) and postnatal (right) age for males and females.

R1#22: Clarify expressions: 'relay weak sensory drive,' 'how translating intracortical microstructure profiling,' 'transfer developmental control from genetic preprogramming to increasing experience-dependent plasticity.'

We have taken the Reviewer's suggestion into consideration and clarified some of these expressions in the text of our manuscript. The sentence referring to "relay weak sensory drive" has been reframed, whereas the sentence referring to "transfer developmental control from genetic preprogramming to increasing experience-dependent plasticity" has been removed.

Introduction (P.3, L.41-43)

"Neural activity progressively refines this protomap, shifting from predominately spontaneous synchronised bursts during gestation to more sensory-driven thalamocrortical activity postnatally (Molnar et al., 2020)."

Discussion (P.27, L.426-428)

"In conclusion, the present study demonstrates how intracortical profiling of neonatal MRI can reveal distinct spatial patterns of development, which reflect the regional differentiation across large-scale spatial gradients."

R1#23: 'long-term cognitive vulnerability' preterm infants tend to be affected across multiple domains, including cognitive outcomes, would neurodevelopmental vulnerability be more encompassing?

We thank the Reviewer for this meaningful suggestion. This part of the Introduction has been rephrased in the revised manuscript.

Introduction (P.3, L.51-53)

"In addition, understanding the impact of each developmental period will allow us to gain insight into how premature birth disrupts neurodevelopmental processes, and increases the risk of neurodevelopmental disorders (Gozdas et al., 2018; Smyser et al., 2016)."

R1#24: I am not sure I understand the 'differentiable' in this sentence: 'In contrast, we observed decreases in profile variance during the postnatal period, suggesting that cortical layers become more differentiable in the first postnatal weeks'. In methods you defined variance as 'the spread of intensities across the profile. A higher variance relates to a flatter intensity profile (i.e. uniformly distributed intensities across intracortical depths) and a low variance describes a heterogeneous intensity distribution across intracortical depths'. Decrease in variance thus reflects more uniform distribution (less differentiable laminae), or maybe I have misunderstood your statement?

We understand the confusion with the interpretation of variance, as it contradicts the typical understanding of higher variance = more differentiation. As pointed out by the Reviewer, higher variance in the case of central moments relates to a flatter (i.e. more uniform) profile. Hence, a decrease

in variance means that the profile becomes less flat, or more heterogeneous, thus the layers are more differentiable.

Results (P.20, L.295-297)
"Interestingly, we observed decreases in profile variance, corresponding to a less flat profile, thus suggesting that cortical layers become more differentiable in the first postnatal weeks, in contrast to the increasing homogeneity during prenatal development."

R1#25: The statement that macroscale features 'do not relate to cytoarchitectural changes' is too strong. While coarse, thickness and surface area reflect some cytoarchitectural aspects (laminar thickness, regional cell density). It would be beneficial to soften this wording
We thank the Reviewer for this suggestion. We indeed agree that cortical thickness and surface area reflect some cytoarchitectural aspects and we have therefore softened the statement.

Introduction (P.4, L.58-64)
"However, extant *in vivo* research has primarily been constrained to macroscale features, such as thickness and surface area, which are relatively insensitive to finer-scale features, e.g. cortical columns and layers (la Fougere et al., 2011; Ribeiro et al., 2013). Yet these finer-scale features are progressively refined during perinatal neurodevelopment and lay the foundation for functional specialisation of each cortical region (Meyer et al., 2000; Molnar et al., 2019), therefore there is a need to shift the focus on investigating microstructure rather than morphology alone."

R1#26: 'mesiotemporal axis' - 'mesiotemporal lobe' or do you have something more specific in mind?
We thank the Reviewer for pointing out the typographical mistake. However, in the revised manuscript, has been removed.

R1#27: 'postnatal age of 6+6 weeks' is this std? +-?
For the ages of neonates, it is a convention to write them in weeks with days as superscript, where 6^{+6} represents 6 weeks and 6 days.

Methods (P.8, L.115-116)
"All ages/developmental measures are reported in $\text{weeks}^{+\text{days}}$, e.g. "$6^{+6}$ weeks"."

R1#28: FYI, I tried to provide as much localization in the text as possible for my comments, but without line numbers this was difficult. I would encourage the authors to include them in their future submissions to make the reviewers' lives easier 0:)
We thank the Reviewer for this helpful suggestion. We now added line numbers to the clean version of the manuscript.

R1 conclusion: Thank you for the opportunity to review this interesting work, I hope my comments will be helpful. With clarification of methodological limitations, careful framing of developmental interpretations, and minor revisions to terminology and figures, the manuscript will provide valuable insight into early cortical microstructural development. I wish the authors the best in their revisions.
We appreciate the constructive feedback from the Reviewer. All comments significantly helped to improve the clarity of the manuscript.

Reviewer #2:

R2 summary: The manuscript provides a thorough and rigorous analysis of T1/T2 ratio offering insights into the spatial patterning of microstructure following birth. The results are complemented by a thoughtful summary of early biological mechanisms governing areal patterning of the human cortex. However, the interpretation of the changes in the context of

sensory isolation vs external sensory stimulation from the postnatal environment is a bit oversimplistic. The text would be improved with added nuance to the interpretation of findings.
We would like to sincerely thank Reviewer 2 for their positive evaluation of our work. We welcome their recommendation to discuss the complex interactions of biological programming and external influences with greater nuance and depth.

R2#1: "After birth, aside from the intrinsic refinement of the cortical structure, sensory input drives neuronal patterning." Neonates spend approximately 70% of their time asleep. Given the disproportionate amount of time spent asleep, research suggests that electrical activity during sleep plays a crucial role in the developing motor and visual system (PMCID: PMC4884844; PMCID: PMC9254005).

"In utero the foetus is relatively isolated from sensory input and therefore genetic and epigenetic programming primarily steer neurobiological processes" There is sensory input from maternal voice and heartbeat (PMCID: PMC4364233) and sensory input from motor movements from the infant during sleep (PMID: 26364005) that likely contribute to brain development.

We would like to thank the Reviewer for highlighting these interesting and relevant studies. We updated the text, incorporating these points, to further enhance our Introduction.

Introduction (P.3, L.46-49)
"After birth, alongside internally generated activity (e.g. during sleep), neurodevelopment is increasingly shaped by experience-dependent plasticity, involving reinforcement of frequently used axons through myelination and pruning of infrequently used synapses (Huttenlocher & Dabholkar, 1997; Lokhandwala & Spencer, 2022; Stiles & Jernigan, 2010)."

Introduction (P.3, L.43-46)
"While *in utero* the foetus is sensitive to the maternal heartbeat, touch of the abdomen and external noise (Marx & Nagy, 2015; Webb et al., 2015), genetic programs primarily steer neurodevelopmental processes (Bystron et al., 2008; Webb et al., 2001)."

R2#2: "This modulatory transition highlights the importance of the timing of birth in brain development, with prematurely born infants being exposed to a sensory-rich environment before the scaffold of their circuitry has been completely established for postnatal plasticity." The clinical characteristics of the preterm neonates included in the analysis are not described. However, the study includes extremely preterm and very preterm neonates. Extremely preterm and very preterm neonates often have interventions to sustain life (e.g., mechanical ventilation, antenatal steroids, humidity and oxygenation control, antibiotics). Disruptions to oxygenation and blood flow, and associated brain injury, are more likely the culprit to aberrant grey and white matter development than a 'sensory-rich environment'. Further, disrupted sleep from feeding and for necessary medical interventions is likely a major contributing factor to disrupted brain growth in prematurely born infants.
The Reviewer is correct to point out that other clinical factors rather than solely the earlier exposure to a sensory-rich environment contribute significantly to the disrupted cortical development in extremely and very preterm infants. We have therefore softened our corresponding statements in the Discussion and also acknowledged these clinical factors in the limitations of our study.

Discussion (P.23, L.354-358)
"This modulatory transition highlights the importance of the timing of birth in brain development, as prematurely born infants are exposed to extra-uterine sensory input at a stage when their cortical circuitry is still undergoing rapid organisation, which may contribute to altered trajectories of postnatal plasticity. However, other significant clinical factors, such as mechanical ventilation, antenatal steroids and oxygenation control, are contributing to the disrupted cortical growth of prematurely born infants."

R2#3: "In general, the prominent effect of gestational maturation on cortical microstructure emphasises the importance of the timing of birth and indicates that premature transition to the ex utero environment could disturb the well-regulated developmental trajectories of cortical growth, an insight that aligns with literature across various modalities" It's not clear that the current results demonstrate that premature birth disturbs developmental trajectories. Is there an interaction between postnatal age and GA? i.e., do neonates born extremely or very preterm have a lower postnatal correlation with microstructure intensity than term born neonates?

We thank the Reviewer for this important clarification. We agree that our results don't directly demonstrate that premature birth disturbs developmental trajectories. We tested the interaction between GA and PNA (central moments ~ GA x PNA + sex) and found that this interaction is significant after FDR correction only in 15 parcels when modelling centre of gravity and no parcels when modelling variance. Therefore, we have softened the corresponding sentence in the Discussion of our revised manuscript, now describing that the idea of earlier transition to the ex utero environment influencing typical cortical development is a speculative interpretation consistent with prior literature, rather than a conclusion from our analyses.

Discussion (P.24, L.375-379)

"In general, the prominent effect of gestational maturation on cortical myelin hints toward the importance of the timing of birth and is consistent with literature across various modalities supporting that premature transition to the *ex utero* environment may alter the otherwise well-regulated developmental trajectories of cortical growth (Ball et al., 2013; Dimitrova et al., 2021; Fenn-Moltu et al., 2023; Franca et al., 2024)."

R2#4: The abstract says (n=599, 0-7 weeks). Rather than the postnatal age, it would be more informative to state the GA or PMA.

We replaced the PNA range with the PMA range of the participants of the study.

Abstract (P.1, L.14-17)

"Leveraging submillimetre T1-weighted/T2w-weighted (T1w/T2w) magnetic resonance imaging (MRI) from pre- and full-term neonates (n = 599, 26-44 weeks), we sampled intracortical profiles across the cortex and characterised the profiles' shapes according to their central moments."

R2#5: The x-axis is extremely small font in figures 1-2.

We have adjusted the font of the figures accordingly.

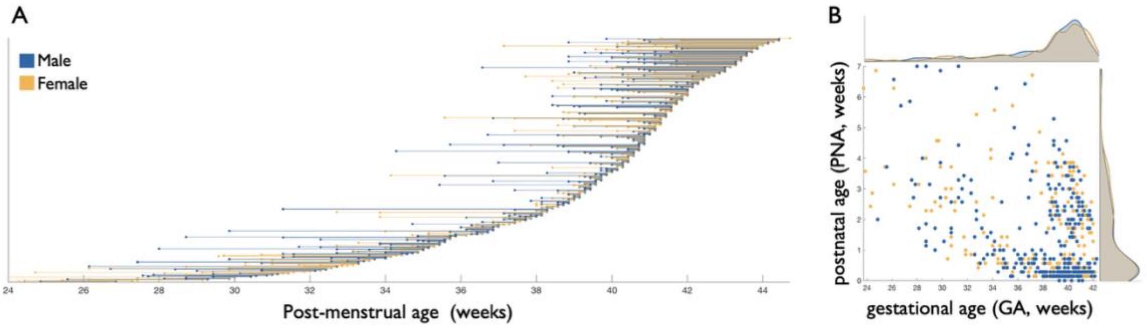

Figure 6: A) Age distributions of the 599 participants (y-axis). The plot…

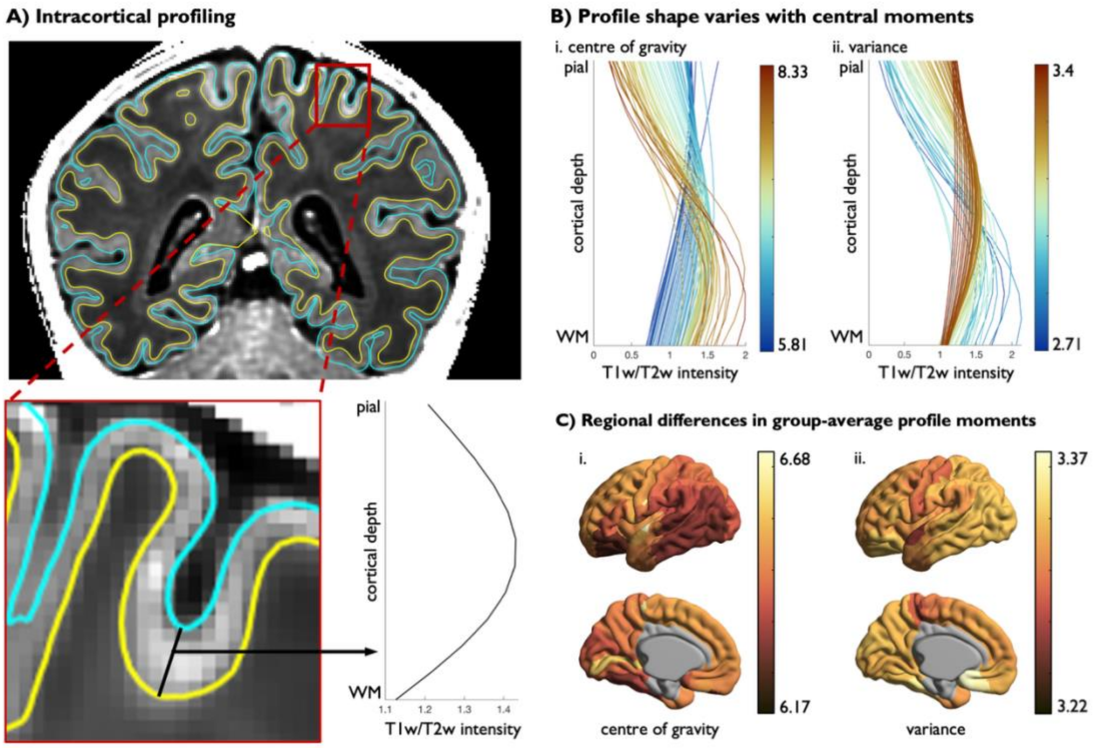

Figure 7: A) Intracortical intensity profiles were extracted at each vertex…

Reviewer #3:

R3 summary: This manuscript investigates how intrinsic and extrinsic factors drive maturation of the human cortex during late gestation and early postnatal development. The authors used T1-weighted and T2-weighted MRI scans from the Developing Human Connectome Project (dHCP), leveraging the T1w/T2w ratio as a biomarker to characterize cortical microstructural changes. To present intracortical microstructure profiles, the neonatal cortex was divided into 12 layers, and the profile shapes were described using central moments inspired by prior histological work. The effects of gestational and postnatal age on these profiles were examined and found to align with low spatial-frequency geometric eigenmodes of the cortex. It is commendable that the authors attempted laminar-level analysis of the neonatal cortex to assess intrinsic and extrinsic factors, and the relatively large dataset of 599 neonates is a notable strength. However, without a strong fundamental basis for dividing the neonatal cortex into 12 layers, and without sufficient justification of the accuracy of values extracted from each layer, the subsequent analyses risk being perceived as a mathematical exercise rather than a substantive contribution to our understanding of cortical development. Such analysis to MRI is fundamentally limited by much lower resolution of MRI compared to that of histological images. It is almost impossible to justify expanding histological framework to MRI data as conducted in this manuscript.

We thank the Reviewer for their comments on the manuscript. We hope to clarify their concerns about the methodology of the study in the following response.

R3#1: The authors constructed 12 equivolumetric surfaces between the pial and white matter surfaces. However, the average human cortex is approximately 2-4 mm thick, and neonatal cortices are even thinner. The raw T1-weighted image resolution is 0.8 x 0.8 x 1.6 mm3. In theory, the images can be upsampled to any higher resolution, but any upsampling is just artificial, not bringing real new information. Dividing the neonatal cortex with acquisition resolution of only 1-2 voxels into 12 layers appears to be just mathematic exercise instead of revealing any validated information. The cited paper (Waehnert et al., 2014) conducted the analysis on ex-vivo images acquired on 7T scanner with resolution of 140 um, which cannot fully justify the same methods could be employed in this study. In the current context, this approach raises concerns about artificial measures, and a high degree of voxel overlap between adjacent layers. Furthermore, the segmentation results shown in Fig. 2A appear incorrect in the central regions of the brain, which

raises additional concerns about segmentation accuracy even before performing laminar analysis. The partial volume effects are big. The deepest cortical layers are likely to be contaminated by white matter voxels, while the most superficial layers may be affected by CSF signals. Finally, no laminar surface mapping figures were presented in the main results or supplementary materials, which further undermines validity in the study's central claim of capturing cortical laminar organization.

We thank the Reviewer for raising these important concerns regarding intracortical profiling in neonatal MRI. We fully agree that the thinness of the neonatal cortex poses a substantial challenge for layer-resolved analyses. Our approach does not aim to delineate cytoarchitectural layers (an impossible feat with data of this resolution), but rather to characterise continuous depth-dependent variations in myelin.

Motivated by similar concerns as those posed by the Reviewer, we recently conducted a set of experiments on the biological validity of in vivo intracortical profiling [(Paquola et al., 2025); https://doi.org/10.1101/2025.10.16.682836]. Using an ultra-high-resolution T1 dataset [0.25 mm isotropic; (Lusebrink et al., 2021)], we examined the impact of downsampling the data on the resultant profiles. While lower-resolution profiles are smoother, the corresponding moment maps remain highly consistent with the original high-resolution data (r > 0.95 at 0.7 mm; r > 0.91 at 1 mm). These findings support that, even at the resolution of our neonatal data, microstructure profiling can capture meaningful, regional variations in laminar organisation.

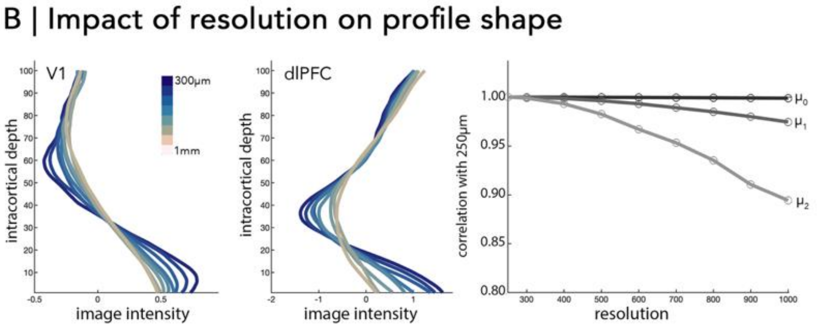

Results from Paquola et al. (2025) showing how central moments derived from lower-resolution imaging recapitulate the patterns observed in higher-resolution, laminar-relevant data.

Key to this approach is oversampling within the cortical ribbon and trilinear interpolation. Together, these preprocessing steps maximise the number of unique intensity values per profile. Central moments then summarise the *shape* of the intracortical signal, which provides more robust metrics compared to depth-specific intensities.

We agree that partial volume with white matter and CSF is unavoidable at this resolution. Nevertheless, in previous work, we've shown that correcting for PVE has little to no impact on the central moments (Paquola et al., 2019a). This is once again an advantage of focussing on the shape of the profiles rather than depth-specific intensities. Furthermore, the GM/WM boundary is not a sharp division even at microscopic scales, and signal mixing in deeper layers may index interesting developmental changes in the long-range projections and the underlying U-fibre system.

Finally, considering the segmentation shown in Figure 2A, we want to clarify that the central part that the Reviewer is likely remarking upon is the medial wall, a non-cortical area of the surface reconstruction that is not included in our analyses.

Methods (P.14, L.210-215)

"A recent study has tested the validity of using central moments in lower-resolution data by downsampling ultra-high-resolution T1 data (Lusebrink et al., 2021; Paquola et al., 2025). The central moment maps of the downsampled data were highly similar to the maps of the original data (r > 0.9), a

finding supporting that, even at the resolution of neonatal data, microstructure profiling can capture meaningful, regional variations in laminar organisation."

Methods (P.15, L.217-227)
"Partial volume effects with white matter are unavoidable at the resolution of the analysed scans, however, previous work has shown that correcting for these effects has little to no impact on the central moments (Paquola et al., 2019a), since these describe the intracortical distribution of intensities instead of depth-specific intensities. Additionally, the boundary between the grey and white matter is not sharply defined, therefore signal-mixing in deeper layers may index interesting developmental changes in long-range projections and the underlying U-fibre system. Together, the equivolumetric spacing, the oversampling using trilinear interpolation, as well as the use of central moments to summarise the intracortical profiles ensure a robust and thickness-insensitive characterisation of intracortical T1w/T2w distributions at the resolution available. This approach enables the reliable investigation of the regional variation of laminar organisation, somewhat overcoming the limitation of the relatively low resolution of the scans."

R3#2: While one of the major highlights in the Methods is "histology-inspired intracortical profiling", it is not clear how the measure -- center of gravity (CoG) was computed in this study, and the cited reference (Schleicher et al., 1999) does not provide a clear corresponding explanation. If this is the first time this measure was employed in the imaging study, more detailed and rigorous explanation or testing of the measure is expected. Figure 2B is particularly confusing: of all three small panels, the x-axis is labeled as T1w/T2w intensity and the y-axis as cortical depth, but it is unclear how the different panels separately represent mean intensity, CoG, and variance. In addition, the meaning of the color coding is not explained—does it represent age or these center measure?

We thank the Reviewer for highlighting the need for clarification. While central moments have been employed previously to summarise intracortical profiles in vivo (Paquola et al., 2019a; Park et al., 2024; Royer et al., 2023), we agree that additional detail on their computation is warranted for clarity. Furthermore, in the updated manuscript, we highlight a recent set of experiments on the validity, reliability and replicability of in vivo intracortical profiles (Paquola et al., 2025).

Methods (P.14, L.199-208)
"Computation of $\mu 1$ and $\mu 2$ involves treating the profile as a histogram with intracortical depths as bins and the intensities as frequency, then simply computing the mean and standard deviation values. Thereby, the first moment, *centre of gravity* (μ_1), describes how the intensity distribution is balanced across cortical depths, whereby a high centre of gravity corresponds to higher intensities in the deeper cortex and a low centre of gravity describes higher intensities in the superficial cortex, hence providing an indicator of the balance of microstructure across supra- and infragranular layers (**Figure 2Bi**). The second moment, *variance* (μ_2), characterises the spread of intensities across the profile. A higher variance relates to a flatter intensity profile (i.e. uniformly distributed intensities across intracortical depths) and a low variance describes a heterogeneous intensity distribution across intracortical depths (**Figure 2Bii**)."

Methods (P.14, L.210-215)
"A recent study has tested the validity of using central moments in lower-resolution data by downsampling ultra-high-resolution T1 data (Lusebrink et al., 2021; Paquola et al., 2025). The central moment maps of the downsampled data were highly similar to the maps of the original data, a finding supporting that, even at the resolution of neonatal data, microstructure profiling can capture meaningful, regional variations in laminar organisation."

We have also adjusted the Figure 2B accordingly, to make the separation of the panels and the colour-coding clearer.

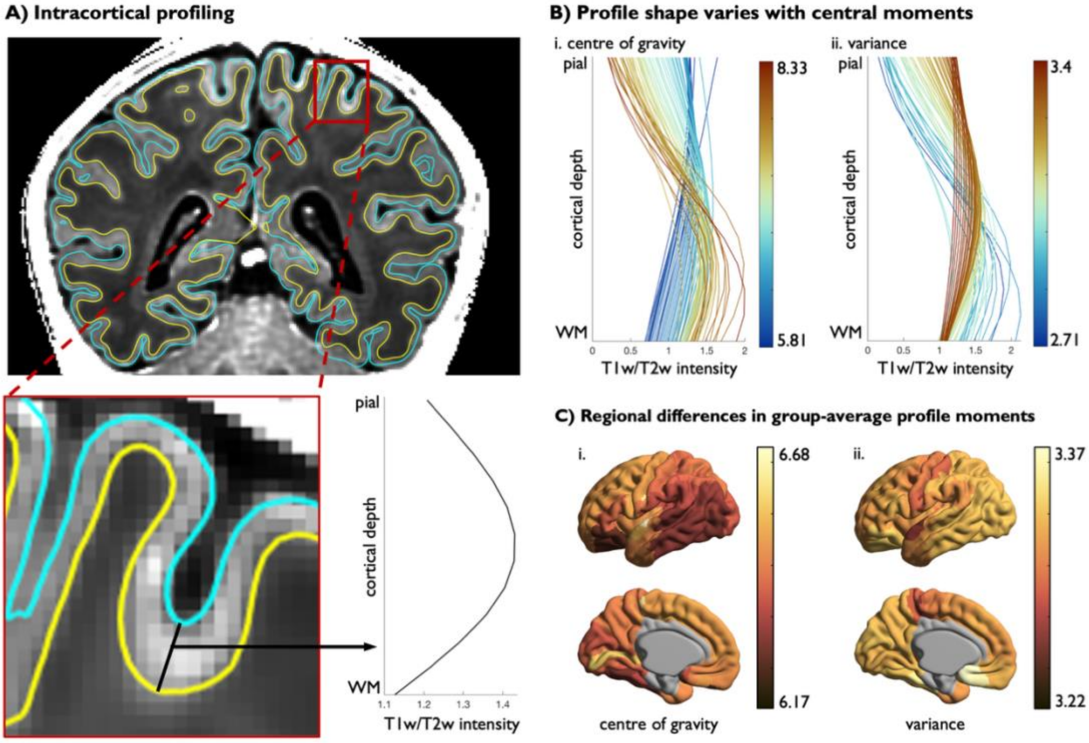

Figure 8: A) Intracortical intensity profiles were extracted at each vertex of infant structural MRI scans. Intensities were sampled at twelve equivolumetric intracortical surfaces, spanning from the pial boundary (blue) to the white matter boundary (yellow), capturing signal variations across cortical depths, defined as microstructure profiles. **B)** The shape of the average microstructure profile within each von Economo parcel was summarised using two central moments: centre of gravity, and variance. **C)** Parcel-wise central moment distributions mapped on the dHCP 40-week surface template. Excluded regions (i.e. von Economo areas L_{A1}, L_{A2}, L_{C1}, L_{C2}, L_{C3}, L_D and L_E and the cortical wall) are shown in grey. Due to the high inter-hemispheric correlation of the central moments (r = 0.79 for centre of gravity and r = 0.93 for variance), we show only results of the left hemisphere for simplicity.

R3#3: The authors also conducted product-moment correlations between the postmenstrual, gestational and postnatal age effects and the 2nd-4th geometric eigenmodes. However, the manuscript does not provide a detailed explanation of how these eigenmodes were computed, and the cited reference (Pang et al., 2023) is missing from the reference list.

We thank the Reviewer for pointing this out. We have updated the corresponding part of the Methods to better explain how the eigenmodes were computed. We also added the cited reference in the reference list.

Methods (P.18, L.253-257)

"Geometric eigenmodes were computed by applying the Laplace-Beltrami operator (LBO) to the surface mesh of a population-average infant neocortex ["dhcpSym" 40-week, (Williams et al., 2023)], using code provided by Pang et al. (2023). The LBO treats the cortical surface as a Riemannian manifold and computes eigenfunctions of the surface Laplacian, capturing geometric patterns from coarse to fine scales."

R3#4: Throughout the manuscript, terms such as "microstructural density," "intracortical homogeneity," and "variance" are sometimes used interchangeably or without precise definition. For readers less familiar with histology-inspired moment metrics, a more consistent phrasing would make the results easier to interpret.

In light of this review and our recent analyses on the reliability of intracortical T1w/T2w [(Paquola et al., 2025); https://doi.org/10.1101/2025.10.16.682836], we made two substantial revisions to the manuscript. First, we removed µ0 (mean intensity) from all analyses and now focus exclusively on µ1 (centre of gravity) and µ2 (variance). Second, we revised our terminology to describe these moments in relation to intracortical myelin rather than using the less precise term "microstructural density".

In the original submission we were cautious about framing T1w/T2w as a myelin marker, largely due to evidence that T1w/T2w does not correspond to inter-regional differences in myelin staining acquired in the same brain (Sandrone et al., 2023). However, our analyses of a multi-modal test-retest dataset (Shams et al., 2019) now show that individual-specific patterns of μ1 and μ2 derived from T1w/T2w and R1 are highly similar ($r = 0.95$) (Paquola et al., 2025). Given R1 is strongly correlated with histological myelin [$r = 0.805$ (Stuber et al., 2014)], this finding strengthens our confidence that these measures, derived from intracortical T1w/T2w, primarily reflect depth-wise variations in myelin. In contrast, μ0 shows substantially weaker correspondence between T1w/T2w and R1. This effect, as well as the limited histological validation reported by Sandrone et al. (2023), likely reflects B1+ transmit-field inhomogeneities, which are known to affect T1w/T2w intensities (Glasser et al., 2022). Higher-order moments (μ1 and μ2) are less susceptible to these biases because they encode relative intensity distributions within a region and are therefore more robust to low-spatial-frequence B1+ variations.

Introduction (P.5, L.66-81)

"Intracortical profiling of sub-millimetre resolution *in vivo* MRI offers a novel opportunity to tap into the micro-scale maturation of the cortex. Recent work has shown that microstructure profiles, extracted from T1-weighted/T2-weighted (T1w/T2w) imaging, capture regional differences in laminar organisation, as indicated by post-mortem histology (Paquola et al., 2025; Paquola et al., 2019b; Sui et al., 2022). T1w/T2w imaging enhances cortical myelin contrast by mathematically cancelling out receive field (B1-) bias (Glasser et al., 2014; Glasser & Van Essen, 2011; Nakamura et al., 2017). The residual transmit field (B1+) biases, however, vary across the field of view and across participants in such a way that absolute differences in T1w/T2w values cannot be directly interpreted as absolute differences in cortical myelin (Glasser et al., 2022; Sandrone et al., 2023). Inspecting relative T1w/T2w levels across cortical depths within an area overcomes this issue, however, due to the low spatial frequency of the B1+ biases (MacLennan et al., 2022) Indeed, using a multi-modal test-retest dataset (Shams et al., 2019), Paquola et al. (2025) recently showed that the shape of T1w/T2w intracortical profiles are reliable and exhibit very similar individual-specific spatial patterns to those derived from quantitative R1. Combined with evidence that R1 is strongly correlated with histological myelin (Stuber et al., 2014), these findings support that the intracortical balance of T1w/T2w effectively proxies depth-wise variations in myelin, and these values can be compared across participants."

Considering the definition of the central moments, we have better explained these terms in our revised manuscript in order to make the interpretation clearer.

Methods (P.14, L.205-208)

"The second moment, *variance* (μ_2), characterises the spread of intensities across the profile. A higher variance relates to a flatter intensity profile with a more homogeneous intensity distribution along depths (i.e. uniformly distributed intensities across intracortical depths) and a low variance describes a heterogeneous intensity distribution across intracortical depths (**Figure 2Bii**)."

References

Ball, G., Srinivasan, L., Aljabar, P., Counsell, S. J., Durighel, G., Hajnal, J. V., Rutherford, M. A., & Edwards, A. D. (2013). Development of cortical microstructure in the preterm human brain. *Proc Natl Acad Sci U S A*, *110*(23), 9541-9546. https://doi.org/10.1073/pnas.1301652110

Bystron, I., Blakemore, C., & Rakic, P. (2008). Development of the human cerebral cortex: Boulder Committee revisited. *Nat Rev Neurosci*, *9*(2), 110-122. https://doi.org/10.1038/nrn2252

Cadwell, C. R., Bhaduri, A., Mostajo-Radji, M. A., Keefe, M. G., & Nowakowski, T. J. (2019). Development and Arealization of the Cerebral Cortex. *Neuron*, *103*(6), 980-1004. https://doi.org/10.1016/j.neuron.2019.07.009

Dimitrova, R., Pietsch, M., Ciarrusta, J., Fitzgibbon, S. P., Williams, L. Z. J., Christiaens, D., Cordero-Grande, L., Batalle, D., Makropoulos, A., Schuh, A., Price, A. N., Hutter, J., Teixeira, R. P., Hughes, E., Chew, A., Falconer, S., Carney, O., Egloff, A., Tournier, J. D.,...O'Muircheartaigh, J. (2021). Preterm birth alters the development of cortical microstructure and morphology at term-equivalent age. *Neuroimage*, *243*, 118488. https://doi.org/10.1016/j.neuroimage.2021.118488

Fenn-Moltu, S., Fitzgibbon, S. P., Ciarrusta, J., Eyre, M., Cordero-Grande, L., Chew, A., Falconer, S., Gale-Grant, O., Harper, N., Dimitrova, R., Vecchiato, K., Fenchel, D., Javed, A., Earl, M., Price, A. N., Hughes, E., Duff, E. P., O'Muircheartaigh, J., Nosarti, C.,...Batalle, D. (2023). Development of neonatal brain functional centrality and alterations associated with preterm birth. *Cereb Cortex*, *33*(9), 5585-5596. https://doi.org/10.1093/cercor/bhac444

Franca, L. G. S., Ciarrusta, J., Gale-Grant, O., Fenn-Moltu, S., Fitzgibbon, S., Chew, A., Falconer, S., Dimitrova, R., Cordero-Grande, L., Price, A. N., Hughes, E., O'Muircheartaigh, J., Duff, E., Tuulari, J. J., Deco, G., Counsell, S. J., Hajnal, J. V., Nosarti, C., Arichi, T.,...Batalle, D. (2024). Neonatal brain dynamic functional connectivity in term and preterm infants and its association with early childhood neurodevelopment. *Nat Commun*, *15*(1), 16. https://doi.org/10.1038/s41467-023-44050-z

Glasser, M. F., Coalson, T. S., Harms, M. P., Xu, J., Baum, G. L., Autio, J. A., Auerbach, E. J., Greve, D. N., Yacoub, E., Van Essen, D. C., Bock, N. A., & Hayashi, T. (2022). Empirical transmit field bias correction of T1w/T2w myelin maps. *Neuroimage*, *258*, 119360. https://doi.org/10.1016/j.neuroimage.2022.119360

Glasser, M. F., Goyal, M. S., Preuss, T. M., Raichle, M. E., & Van Essen, D. C. (2014). Trends and properties of human cerebral cortex: correlations with cortical myelin content. *Neuroimage*, *93 Pt 2*, 165-175. https://doi.org/10.1016/j.neuroimage.2013.03.060

Glasser, M. F., & Van Essen, D. C. (2011). Mapping human cortical areas in vivo based on myelin content as revealed by T1- and T2-weighted MRI. *J Neurosci*, *31*(32), 11597-11616. https://doi.org/10.1523/JNEUROSCI.2180-11.2011

Gozdas, E., Parikh, N. A., Merhar, S. L., Tkach, J. A., He, L., & Holland, S. K. (2018). Altered functional network connectivity in preterm infants: antecedents of cognitive and motor impairments? *Brain Struct Funct*, *223*(8), 3665-3680. https://doi.org/10.1007/s00429-018-1707-0

Huttenlocher, P. R., & Dabholkar, A. S. (1997). Regional differences in synaptogenesis in human cerebral cortex. *J Comp Neurol*, *387*(2), 167-178. https://doi.org/10.1002/(sici)1096-9861(19971020)387:2<167::aid-cne1>3.0.co;2-z

la Fougere, C., Grant, S., Kostikov, A., Schirrmacher, R., Gravel, P., Schipper, H. M., Reader, A., Evans, A., & Thiel, A. (2011). Where in-vivo imaging meets cytoarchitectonics: the relationship between cortical thickness and neuronal density measured with high-resolution [18F]flumazenil-PET. *Neuroimage*, *56*(3), 951-960. https://doi.org/10.1016/j.neuroimage.2010.11.015

Lariviere, S., Vos de Wael, R., Hong, S. J., Paquola, C., Tavakol, S., Lowe, A. J., Schrader, D. V., & Bernhardt, B. C. (2020). Multiscale Structure-Function Gradients in the Neonatal Connectome. *Cereb Cortex*, *30*(1), 47-58. https://doi.org/10.1093/cercor/bhz069

Lokhandwala, S., & Spencer, R. M. C. (2022). Relations between sleep patterns early in life and brain development: A review. *Dev Cogn Neurosci*, *56*, 101130. https://doi.org/10.1016/j.dcn.2022.101130

Lusebrink, F., Mattern, H., Yakupov, R., Acosta-Cabronero, J., Ashtarayeh, M., Oeltze-Jafra, S., & Speck, O. (2021). Comprehensive ultrahigh resolution whole brain in vivo MRI dataset as a human phantom. *Sci Data*, *8*(1), 138. https://doi.org/10.1038/s41597-021-00923-w

MacLennan, T., Seres, P., Rickard, J., Stolz, E., Beaulieu, C., & Wilman, A. H. (2022). Characterization of B(1)(+) field variation in brain at 3 T using 385 healthy individuals across the lifespan. *Magn Reson Med*, *87*(2), 960-971. https://doi.org/10.1002/mrm.29011

Marx, V., & Nagy, E. (2015). Fetal Behavioural Responses to Maternal Voice and Touch. *PLoS One*, *10*(6), e0129118. https://doi.org/10.1371/journal.pone.0129118

Meyer, G., Schaaps, J. P., Moreau, L., & Goffinet, A. M. (2000). Embryonic and early fetal development of the human neocortex. *J Neurosci*, *20*(5), 1858-1868. https://doi.org/10.1523/JNEUROSCI.20-05-01858.2000

Molnar, Z., Clowry, G. J., Sestan, N., Alzu'bi, A., Bakken, T., Hevner, R. F., Huppi, P. S., Kostovic, I., Rakic, P., Anton, E. S., Edwards, D., Garcez, P., Hoerder-Suabedissen, A., & Kriegstein, A. (2019). New insights into the development of the human cerebral cortex. *J Anat*, *235*(3), 432-451. https://doi.org/10.1111/joa.13055

Molnar, Z., & Kwan, K. Y. (2024). Development and Evolution of Thalamocortical Connectivity. *Cold Spring Harb Perspect Biol*, *16*(1). https://doi.org/10.1101/cshperspect.a041503

Molnar, Z., Luhmann, H. J., & Kanold, P. O. (2020). Transient cortical circuits match spontaneous and sensory-driven activity during development. *Science*, *370*(6514). https://doi.org/10.1126/science.abb2153

Nakamura, K., Chen, J. T., Ontaneda, D., Fox, R. J., & Trapp, B. D. (2017). T1-/T2-weighted ratio differs in demyelinated cortex in multiple sclerosis. *Ann Neurol*, *82*(4), 635-639. https://doi.org/10.1002/ana.25019

Pang, J. C., Aquino, K. M., Oldehinkel, M., Robinson, P. A., Fulcher, B. D., Breakspear, M., & Fornito, A. (2023). Geometric constraints on human brain function. *Nature*, *618*(7965), 566-574. https://doi.org/10.1038/s41586-023-06098-1

Paquola, C., Bethlehem, R. A., Seidlitz, J., Wagstyl, K., Romero-Garcia, R., Whitaker, K. J., Vos de Wael, R., Williams, G. B., Consortium, N., Vertes, P. E., Margulies, D. S.,

Bernhardt, B., & Bullmore, E. T. (2019a). Shifts in myeloarchitecture characterise adolescent development of cortical gradients. *Elife*, *8*. https://doi.org/10.7554/eLife.50482

Paquola, C., Royer, J., Tsigaras, T., Cabalo, D. G., Hwang, Y., Hoffstaedter, F., Eickhoff, S. B., & Bernhardt, B. C. (2025). Intracortical microstructure profiling: a versatile method for indexing cortical lamination. *bioRxiv*, 2025.2010.2016.682836. https://doi.org/10.1101/2025.10.16.682836

Paquola, C., Vos De Wael, R., Wagstyl, K., Bethlehem, R. A. I., Hong, S. J., Seidlitz, J., Bullmore, E. T., Evans, A. C., Misic, B., Margulies, D. S., Smallwood, J., & Bernhardt, B. C. (2019b). Microstructural and functional gradients are increasingly dissociated in transmodal cortices. *PLoS Biol*, *17*(5), e3000284. https://doi.org/10.1371/journal.pbio.3000284

Park, Y., Lee, M. J., Yoo, S., Kim, C. Y., Namgung, J. Y., Park, Y., Park, H., Lee, E. C., Yoon, Y. D., Paquola, C., Bernhardt, B. C., & Park, B. Y. (2024). GAN-MAT: Generative adversarial network-based microstructural profile covariance analysis toolbox. *Neuroimage*, *291*, 120595. https://doi.org/10.1016/j.neuroimage.2024.120595

Ribeiro, P. F., Ventura-Antunes, L., Gabi, M., Mota, B., Grinberg, L. T., Farfel, J. M., Ferretti-Rebustini, R. E., Leite, R. E., Filho, W. J., & Herculano-Houzel, S. (2013). The human cerebral cortex is neither one nor many: neuronal distribution reveals two quantitatively different zones in the gray matter, three in the white matter, and explains local variations in cortical folding. *Front Neuroanat*, *7*, 28. https://doi.org/10.3389/fnana.2013.00028

Royer, J., Lariviere, S., Rodriguez-Cruces, R., Cabalo, D. G., Tavakol, S., Auer, H., Ngo, A., Park, B. Y., Paquola, C., Smallwood, J., Jefferies, E., Caciagli, L., Bernasconi, A., Bernasconi, N., Frauscher, B., & Bernhardt, B. C. (2023). Cortical microstructural gradients capture memory network reorganization in temporal lobe epilepsy. *Brain*, *146*(9), 3923-3937. https://doi.org/10.1093/brain/awad125

Sandrone, S., Aiello, M., Cavaliere, C., Thiebaut de Schotten, M., Reimann, K., Troakes, C., Bodi, I., Lacerda, L., Monti, S., Murphy, D., Geyer, S., Catani, M., & Dell'Acqua, F. (2023). Mapping myelin in white matter with T1-weighted/T2-weighted maps: discrepancy with histology and other myelin MRI measures. *Brain Struct Funct*, *228*(2), 525-535. https://doi.org/10.1007/s00429-022-02600-z

Shams, Z., Norris, D. G., & Marques, J. P. (2019). A comparison of in vivo MRI based cortical myelin mapping using T1w/T2w and R1 mapping at 3T. *PLoS One*, *14*(7), e0218089. https://doi.org/10.1371/journal.pone.0218089

Smyser, C. D., Snyder, A. Z., Shimony, J. S., Mitra, A., Inder, T. E., & Neil, J. J. (2016). Resting-State Network Complexity and Magnitude Are Reduced in Prematurely Born Infants. *Cereb Cortex*, *26*(1), 322-333. https://doi.org/10.1093/cercor/bhu251

Stiles, J., & Jernigan, T. L. (2010). The basics of brain development. *Neuropsychol Rev*, *20*(4), 327-348. https://doi.org/10.1007/s11065-010-9148-4

Stuber, C., Morawski, M., Schafer, A., Labadie, C., Wahnert, M., Leuze, C., Streicher, M., Barapatre, N., Reimann, K., Geyer, S., Spemann, D., & Turner, R. (2014). Myelin and iron concentration in the human brain: a quantitative study of MRI contrast. *Neuroimage*, *93 Pt 1*, 95-106. https://doi.org/10.1016/j.neuroimage.2014.02.026

Sui, Y. V., Masurkar, A. V., Rusinek, H., Reisberg, B., & Lazar, M. (2022). Cortical myelin profile variations in healthy aging brain: A T1w/T2w ratio study. *Neuroimage*, *264*, 119743. https://doi.org/10.1016/j.neuroimage.2022.119743

Webb, A. R., Heller, H. T., Benson, C. B., & Lahav, A. (2015). Mother's voice and heartbeat sounds elicit auditory plasticity in the human brain before full gestation. *Proc Natl Acad Sci U S A*, *112*(10), 3152-3157. https://doi.org/10.1073/pnas.1414924112

Webb, S. J., Monk, C. S., & Nelson, C. A. (2001). Mechanisms of postnatal neurobiological development: implications for human development. *Dev Neuropsychol*, *19*(2), 147-171. https://doi.org/10.1207/S15326942DN1902_2

Williams, L. Z. J., Fitzgibbon, S. P., Bozek, J., Winkler, A. M., Dimitrova, R., Poppe, T., Schuh, A., Makropoulos, A., Cupitt, J., O'Muircheartaigh, J., Duff, E. P., Cordero-Grande, L., Price, A. N., Hajnal, J. V., Rueckert, D., Smith, S. M., Edwards, A. D., & Robinson, E. C. (2023). Structural and functional asymmetry of the neonatal cerebral cortex. *Nat Hum Behav*, *7*(6), 942-955. https://doi.org/10.1038/s41562-023-01542-8
